# Occupational Therapy Neurorehabilitation Practice in Middle East and North Africa (MENA): A Scoping Review

**DOI:** 10.1155/oti/5266195

**Published:** 2026-05-20

**Authors:** Abdulrahman Abdulaziz Abdulrahman Bin-Juwayr, Jacqueline Wesson, Anne Cusick

**Affiliations:** ^1^ Faculty of Medicine and Health, The University of Sydney, Sydney, Australia, sydney.edu.au; ^2^ Faculty of Medical Rehabilitation Sciences, King Abdulaziz University, Jeddah, Saudi Arabia, kau.edu.sa

**Keywords:** cognition, Middle East, neurological rehabilitation, Northern Africa, occupational therapy

## Abstract

**Objective:**

This scoping review aims to map published research evidence about occupational therapy (OT) neurorehabilitation practice in the Middle East North Africa (MENA) region. A secondary aim was to identify what, if any, functional cognition assessments were used by occupational therapists (OTs).

**Methods:**

Following JBI scoping review guidelines, a structured search was conducted across five databases: CINAHL, Embase, Medline, Scopus and Web of Science, concluding in July 2025. Citation chaining expanded the search. Screening and data extraction were managed using Covidence. Descriptive tables and narrative synthesis summarised findings.

**Results:**

Sixty‐seven studies met the inclusion criteria. Most (71.6%) were published in the last decade, mostly in high‐ranking journals. Iran, Israel and Saudi Arabia accounted for 80.5% of studies, and OTs were first or last authors in 47.7%. Services were mainly hospital based (inpatient 47.7%, outpatient 46%), with 59.7% involving multidisciplinary teams. Stroke dominated diagnoses (64.8%), whereas dementia was the least (1.3%). Intervention effectiveness was addressed in 35.8% of studies, including 16.4% randomised controlled trials. Home visits were rarely used. Sixty‐nine standardised assessments were reported, most commonly FIM, FM‐UE, BI, COPM, MMSE, MoCA and LOTCA. No home safety assessments were identified. Only two functional cognition tools were identified, with limited evidence of translation or cultural adaptation. Narrative synthesis revealed that OT neurorehabilitation research in MENA is growing. This aligns with the expansion of WFOT‐accredited OT programmes in MENA, which has increased research output from university‐affiliated OTs.

**Conclusion:**

This study revealed an emerging and rapidly expanding evidence base about OT neurorehabilitation in MENA. It highlights the need for more research about OT for conditions other than stroke. It shows that most research evidence is generated by university‐affiliated OTs, and more practitioner and practice research could be done, for example, home visits. Research about functional cognition is limited, and further investigation about awareness and use is needed.

## 1. Introduction

According to the World Health Organisation (WHO) (2024), more than one‐third of the global adult population is affected by neurological conditions, which are the primary cause of illness and disability worldwide, affecting over three billion people [1]. The prevalence of neurological conditions has led to an increase in disabilities, and providing health and social services for these people has added financial pressure to national healthcare systems, especially in regions with limited resources [2–5]. The Middle East and North Africa (MENA) are one such region. Typically, neurological conditions include stroke, traumatic brain injury (TBI), multiple sclerosis (MS), spinal cord injury (SCI) or dementia [6]. Accordingly, this scoping review focuses exclusively on adult populations who acquired those conditions. Neurorehabilitation is a multidisciplinary intervention that aims to enhance functional recovery and improve quality of life following neurological damage arising from disease, trauma or other disorders [7]. Occupational therapists (OTs) are members of multidisciplinary neurorehabilitation teams, working to enhance functional outcomes and quality of life [8]. The need for multidisciplinary team (MDT) neurorehabilitation is particularly urgent in the MENA region because neurological conditions there are increasing faster than the global average [9]. Neurological conditions are a significant societal concern within the MENA region. As an example, dementia correlates with rising levels of stress and financial strain on caregivers and the absence of formal support systems affecting families and communities across the region [10]. The societal repercussions of the increasing prevalence of neurological conditions are a decline in formal workforce participation, increased reliance on informal caregiving and increased demands on the health system [11, 12].

Interdisciplinary research across neurology, public health and social science provides essential context for understanding neurological conditions in the MENA region. Epidemiological studies have shown the burdens of stroke, migraine and dementia to be largely attributable to modifiable risk factors [9]. Integrated and rehabilitative approaches have been shown to be effective for regionally focused studies that show the impact of population aging and total disability‐adjusted life years (DALYs) due to strokes [13]. The insufficient supply of specialised neurological services is a reason that MENA countries, and many other countries, including those with low and middle incomes, are still struggling with the rising burden of neurological conditions [1]. Along with research on dementia, the extensive caregiving involved and the sociocultural challenges families face present a multifaceted case of how functional cognition is positioned within the routines of daily living [10]. These interdisciplinary insights reinforce the importance of OT frameworks, epidemiological studies, sociocultural analyses and cognitive sciences in understanding and addressing rehabilitation needs.

OTs play a critical role in neurorehabilitation by promoting functional independence and participation in meaningful daily activities. For example, in strokes, cognitive, physical and behavioural difficulties often hinder people′s capacity to carry out daily occupations [8, 14]. To tackle these issues, OTs implement interventions by assessing functional cognition, activity performance and cognitive–environmental variables and employing strategies such as task modification, environmental adjustments and caregiver education [15]. This occupation‐focused approach is important for facilitating reintegration into daily life and reducing long‐term disability, underscoring the need to investigate this topic to inform evidence‐based OT practice [16].

MENA is a region recognised by the WHO, comprising 20 countries, including Saudi Arabia, the United Arab Emirates (UAE), Qatar, Bahrain, Kuwait, Oman, Yemen, Iran, Iraq, Jordan, Syria, Lebanon, Palestine, Israel, Egypt, Algeria, Tunisia, Libya, Mauritania and Morocco [17]. The MENA region has a population of approximately 813 million people [18]. Although mortality from neurological conditions is decreasing in the MENA region, the number of people living with these conditions is rising dramatically, leading to an overall increase in DALYs [9]. Neurological conditions represent a major source of disease burden in the MENA region and were the second leading cause of DALYs after cardiovascular diseases between 1990 and 2019 [9]. More recent Global Burden of Disease (GBD) estimates show that years lived with disability (YLDs) from neurological conditions increased by approximately 22% between 1990 and 2021, with stroke accounting for the largest share of neurological disability [19]. Given this substantial burden and the long‐term disabilities that often result, the demand for comprehensive rehabilitation services is increasing worldwide [20]. In the MENA region, however, a shortage of rehabilitation services persists, alongside a growing demand [9, 14].

As a key member of multidisciplinary neurorehabilitation teams, OTs use remedial and compensatory techniques to ‘address underlying physical, cognitive, and/or visual and perceptual skills to facilitate improved performance and safety during engagement in meaningful and realistic occupations’ ([21], P.135). Despite the need for OTs, there are shortages in the occupational therapy (OT) workforce in MENA [22]. The World Federation of Occupational Therapists (WFOT) identifies that 10 of the 20 countries in the MENA region are members of the WFOT [23]; eight are full members, and two are associate members. To be eligible for full membership, countries must have a national OT association, at least one approved OT education programme and meet WFOT′s standards for professional practice and governance [24]. Associate membership does not require a WFOT‐accredited programme [25]. It is evident from WFOT membership that almost half of the MENA countries have no local OT training opportunities, despite workforce demands [17] and the increasing need for multidisciplinary rehabilitation [12], including rehabilitation for neurological conditions [8, 11]. Currently, limited information is available regarding OT practice in the MENA region. To date, there have been reviews in the MENA region about OT telerehabilitation practice [26] and the use of assistive technology with children with disabilities [27]. No review of evidence on OT neurorehabilitation practice in the MENA region has been conducted. The present study aims to address that gap.

In addition to mapping evidence about OT neurorehabilitation practice, this review will take a particular focus to explore what is known about OT assessment of cognitive impairment and functional cognition in people with neurological conditions in MENA. Cognitive impairments can affect people′s ability to function in activities of daily living (ADLs) and/or instrumental activities of daily living (IADLs) [15]. Understanding cognitive strengths and weaknesses that impact performance is essential in risk assessment and setting recovery and rehabilitation goals [15].

OTs conduct cognitive assessments to determine the impact that cognitive processes, such as memory, attention, problem‐solving and executive functions, may have on ADLs and IADLs [21]. *In addition to these domain-specific cognitive components, functional cognition adds an essential perspective by examining how individuals integrate these cognitive abilities within real world [* 28 *].* This review will explore what cognition assessments are reported in OT neurorehabilitation research, whether or not and how they have been translated and culturally adapted, and if they encompass the newly emerging concept of ‘functional cognition’.

Recent research evidence has shown that traditional question–answer tests used by clinicians, including OTs, such as the Mini‐Mental State Examination (MMSE) [29], are not effective in detecting cognitive impairments that may impact everyday functioning [30]. Functional cognition generally refers to how people use their cognitive abilities to perform real‐world tasks and activities [28, 31, 32], and assessments help OTs understand how cognitive impairments might impact people′s ability to function independently and safely in everyday life [33]. These assessments provide essential information that can be used to set goals and tailor interventions to improve performance in complex daily tasks such as managing finances or meal preparation [28]. Additionally, OT interventions that address functional cognition can improve outcomes; for example, greater investment in OT services focused on functional cognition has been linked to significantly lower hospital readmission rates [34]. Functional cognition is thus an important focus for OT neurorehabilitation practice [31].

No review of OT neurorehabilitation practice in MENA would be complete without consideration of culture. Culture profoundly influences how clients engage with therapy, interpret health and perform ADLs [16]. In OT and rehabilitation, the cultural adaptation of assessments is increasingly recognised as essential for providing equitable and effective care [35–37]. OT must adapt their assessment tools and intervention strategies to clients′ cultural contexts [36, 37], which involves being aware of and responsive to clients′ cultural values, routines and beliefs. In some MENA contexts, rehabilitation practice is shaped by collectivist norms that emphasise family responsibility and shared decision‐making. Studies from Jordan and Oman show that clients often expect family assistance with daily activities, may resist independence‐focused goals and commonly engage in rehabilitation decisions collectively rather than individually [38, 39]. Such practices can conflict with western OT models that prioritise individual autonomy and independence, assumptions that are increasingly critiqued as culturally specific rather than universal [36, 40]. This awareness is necessary to meet client needs, reduce health disparities and improve outcomes. Many widely used OT assessments were developed in English‐speaking western contexts and embed context‐specific assumptions. For example, privileging independence in self‐care, which means that using them without adaptation that reflects MENA cultural norms and practices, is potentially problematic, leading to reduced validity, cultural dissonance and issues in the feasibility of implementation [38]. Cultural relevance must be assessed and, where necessary, adapted in assessments and treatments rather than assumed [38, 40].

This review will characterise the neurorehabilitation practice and research in the MENA region using published research evidence. It will identify where OT neurorehabilitation is practised, the gender of patients and practitioners, what, if any, adaptations have been made to assessments used and if cultural issues have been identified in practice approaches or treatments. Information about the publications and study characteristics will also be included in the review. A secondary aim is to summarise what evidence reveals about cognition and functional cognition assessment in MENA neurorehabilitation. The review will highlight cultural considerations relevant to OT in MENA.

## 2. Materials and Methods

This scoping review was conducted in accordance with the JBI methodology [41] and was registered in the Open Science Framework (OSF) on 24 August 2024 (10.17605/OSF.IO/69E7G), and an a priori protocol was used. No deviation or amendment from the protocol was made. The preliminary search strategy was developed by reading articles related to the topic of MENA, neurorehabilitation and OT presented in the introduction section of this article. Relevant terms used in these articles were identified and mapped to the Medical Subject Headings (MeSH) system. The full search strategy aimed to locate only published studies and was developed in consultation with a medical librarian for Web of Science, Scopus, Medline, Embase and CINAHL from inception to the end of August 2024, with the search repeated for any new sources in mid‐July 2025. Terms were adapted to the controlled vocabularies of each database. Table 1 presents the search strategy for Medline; others are available on request. Citation chaining was used to identify further published sources; the reference lists of two reviews identified in the search, as well as the reference lists of all included sources, were inspected. Sources with relevant titles were identified, located and included in the title–abstract screening. If eligible, they were included in the full‐text screening. All sources eligible for full‐text screening were retrieved.

**Table 1:** Medline search strategy.

Ovid MEDLINE(R) ALL<1946 to 27 August 2024>		
1	Occupational therapy/or occupational therapists/	15817
2	Occupational therap∗.af.	50379
3	OT.tw.	22166
4	1 or 2 or 3	71378
5	Neurological rehabilitation/or cognitive training/or stroke rehabilitation/	20358
6	(Neurological rehab∗ or neurorehab∗ or neural rehab∗ or brain injur∗ rehab∗ or Cognitive∗ train∗ or cognitive rehab∗ or stroke rehab∗).mp.	33981
7	5 or 6	33981
8	Exp “Middle Eastern and North Africans”/or Mauritania/or exp Africa, Northern/or exp Middle East/	212630
9	(“Middle East and North Africa” or Middle East or Saudi Arabia or Bahrain or Iran or Iraq or Yemen or Israel or Kuwait or United Arab Emirates or UAE or Jordan or Lebanon or Oman or Qatar or Syria or Turkey or Northern Africa or Algeria or Egypt or Libya or Morocco or North Africa or Tunisia or Mauritania or MENA countries or MENA region∗).mp.	335815
10	8 or 9	344566
11	4 and 7 and 10	28

Following the search of each database, sources were exported into EndNote Version 21 citation manager [42] and then imported into Covidence [43], a web‐based collaboration software platform. Duplicates were automatically removed by Covidence and manual inspection during title/abstract screening. To be eligible for inclusion, sources needed to be in English, Arabic or French—these are the languages the authors could read and understand; published and peer‐reviewed; in one or more of the MENA countries (identified by study site, named service setting and the affiliation of primary and/or last author); study samples or caseloads that included > 50% adults with primary neurological diagnoses; studies or services that included an OT assessment, intervention or other OT‐related services and finally OTs were involved as participants and/or assessors and/or interventionists and/or authors and/or health professionals in the service. Sources without one or more of these characteristics were excluded. In addition, assessments used for medical diagnostics (e.g., MRI, ultrasound or genetic testing) were excluded. These criteria were used to screen sources first in the title–abstract screen and then in the full‐text screen. Two authors (deidentified‐for‐review) independently screened all titles and abstracts of search results; disagreements were identified in the Covidence platform. A few conflicts were resolved by consensus in discussion with a third author (deidentified‐for‐review). All sources requiring full‐text screening were reviewed by the first author (deidentified‐for‐review), with independent review by one or other authors (deidentified‐for‐review). Disagreements were resolved through consensus discussion by the reviewer pair. Reasons for full‐text exclusion were recorded in the Covidence platform.

All full‐text inclusions were retrieved for data extraction. A data extraction form was developed within Covidence. Extracted data included publication characteristics, study aim, design, demographic and clinical details about the participants, practice or research context, neurorehabilitation service provided, what OTs did in the study (their service or study role), standardised assessments identified, treatments described and the author reported findings that were relevant to the aims of this scoping review. Data extraction and checking (which included confirmation, augmentation or amendment by consensus) were performed as follows: (deidentified‐for‐review) 61 studies extracted, six studies checked; (de‐identified‐for‐review) five checked; and (deidentified‐for‐review) six studies extracted, 61 checked. Any sources that were reviews or systematic review studies screened at the title–abstract stage were inspected to identify original studies not already included. The reviews themselves were not included in the full‐text stage. Relevant citations were tracked, obtained and put into the abstract–title screening pool. These and other sources found through citation chaining of included sources were retrieved, subjected to title–abstract screening, full‐text review and, if eligible, data extraction. A list of all studies excluded at full‐text screen was compiled, with reasons for exclusion noted in Covidence. Extracted data were summarised in a table of all included studies. The characteristics extracted are listed in Tables 2, 3, 4 and 5. The list of all standardised assessments used in the studies is presented in Table 6, listed in order of frequency. The classification of assessment as either a cognitive or a functional cognition was determined using definitions from published references [32, 44–46]. A narrative synthesis of findings was prepared to address review objectives.

**Table 2:** Characteristics of included *n* = 67 MENA studies.

Characteristic (*n* = 67)	*n* (%)
Design	
Cross‐sectional	15 (22.3)
Cohort (prospective, retrospective and longitudinal)	11 (16.4)
Randomised control trial (RCT)	11 (16.4)
Qualitative (semi‐structure‐focus group)	10 (14.9)
Other designs^a^	8 (11.9)
Interventional nonrandomised	8 (11.9)
Multimethod	4 (5.9)
Country	
Iran	23 (34.3)
Israel	21 (31.3)
Saudi Arabia	10 (14.9)
Jordan	3 (4.4)
Kuwait	3 (4.4)
Qatar	2 (2.9)
Egypt	1 (1.4)
Lebanon	1 (1.4)
Morocco	1 (1.4)
Oman	1 (1.4)
United Arab Emirates (UAE)	1 (1.4)
Tunisia, Algeria, Libya, Syria, Yemen, Palestine, Iraq, Bharain and Mauritania	0 (0)
Neurological conditions^b^	
Stroke	48 (64.8)
Other conditions^c^	11 (14.8)
Traumatic brain injury (TBI)	6 (8.1)
Multiple sclerosis (MS)	4 (5.4)
Spinal cord injury (SCI)	4 (5.4)
Dementia	1 (1.3)
Year of publication	
1995–2004	8 (11.9)
2005–2014	11 (16.4)
2015–2025	48 (71.6)

^a^Expert opinion (*n* = 4), economic evaluation (*n* = 2), case report (*n* = 1) and guidelines (*n* = 1).

^b^Some studies included more than one neurological condition. In this table, the numbers represent how frequently each condition was mentioned across all studies, not the number of individual studies.

^c^Patients with disability, mild cognitive impairments, neurological conditions, general, psychiatry cases, brain tumours (glioma and meningioma), encephalitis‐West Nile fever (WNF), Woodhouse–Sakati syndrome and post–COVID‐19 (including neurological sequelae).

**Table 3:** Interventional designs (RCTs, nonrandomised and cohort designs).

Author Year *Country* Journal ranking Question type	Aim	Condition Sample size (*n*) Intervention group (IG) Control group (CG)	Age (years) (mean [SD], range) Sex (F [*n*], %; M [*n*], %)	Context (setting)	^a^Standardised assessments used Outcome measures across time: T1: (baseline) T2: (postintervention) T3: (follow‐up) Or descriptive measure	Intervention overview	OT role(s) ^b^R (researcher) I (interventionist) A (assessor) O (other)	^c^Outcome
Intervention: RCTs design (*n* = 11)								
Carmeli, et al. 2011 *Israel* Q2 [47] *Interventional*	Compare HandTutor + rehab vs. rehab alone for poststroke UE recovery.	Stroke *n* = 31 IG: 16 CG: 15	IG: (57.8 [8.9], NR) CG: (62.5 [5], NR) IG: (F: *n* = 5, 31.25%; M: *n* = 11, 68.75%) CG: (F: *n* = 4, 26.66%; M: *n* = 11, 73.33%)	University and military hospital (inpatient)	All used for T1, T2 and T3: 1. B&B 2. FM	IG: Traditional OT + HandTutor CG: Traditional OT only Frequency (both groups): 20–30 min/day, 5×/week	R: Not OT I: OT delivered both traditional therapy A: Not specified O: Nill	IG showed significant improvement in FM, sensory‐motor ability and tracking from T1 to T3 compared to CG.
Eliav, et al. 2024 *Israel* Q1 [48] *Interventional*	To test I‐PEX′s efficacy for improving EFs in adults with moderate–severe in inpatient traumatic brain injury, and assess its effects on self‐awareness, cognitive self‐efficacy, participation and 1‐month QoL, and inform a large trial.	Traumatic brain injury *n* = 25 IG: 13 CG: 12	IG: (32.31 [10.20], NR) CG: (42.25 [16.91], NR) IG: (F: *n* = 1, 7.7%; M: *n* = 12, 92%) CG: (F: *n* = 3, 25%; M: *n* = 9, 75%)	Subacute inpatient rehabilitation (inpatient)	T1 and T2: 1. LOTCA 2. WebNeuro 3. MET‐HV 4. SPIRQ T3: 5. MPAI‐4 6. QOLIBRI	IG: I‐PEX (module‐based EF training using real‐life inpatient tasks + metacognitive strategies) CG: Treatment‐as‐usual cognitive OT (computer/paper/simulated tasks, strategy feedback). Frequency: 45 min, 3–5×/week, 3–6 weeks	R: First and last authors I: Provided I‐PEX and treatment‐as‐usual A: Conducted cognitive assessment at T1 and T2 O: Nill	MET‐HV (total score): IG improved more than CG from T1 to T2. MET‐HV (completion time): Both groups improved from T1 to T2. WebNeuro EF tasks: IG and CG improved from T1 to T2. SPIRQ: Both groups improved from T1 to T2.
Fawaz, et al. 2023 *Egypt* Q4 [49] *Interventional*	Evaluate repetitive peripheral magnetic stimulation (rPMS) effects on UE function in subacute vs. chronic stroke patients.	Stroke *n* = 80 IG: 40 CG: 40 IG and CG were equally subdivided into subacute (6–24 weeks) and chronic (> 24 weeks) patients.	Total sample: (57.3 [10.7], 18–75) (F: *n* = 24, 30%; M: *n* = 56, 70%)	NR (outpatient clinic)	All used for T1 and T2: 1. FIM 2. FM‐UE 3. AROM (goniometer) 4. MAS	IG: rPMS to abductors, elbow extensors, wrist extensors and the supinator muscle at 30 Hz. CG: Sham rPMS Frequency for both groups (30 min/day, 5 ×/week, 3 weeks), followed by 40 min OT session.	R: Not OT I: OT provided sessions to both groups A: Not OT O: Nill	IG: Improved in FIM, MAS and FM‐UE. Both groups improved in proximal AROM. Subacute in both groups improved more than chronic groups. Mild to moderate (FM‐UE–based subgroups) impairment showed significant gains in AROM.
Givon Schaham, et al. 2024 *Israel* Q2 [50] *Interventional*	To evaluate whether Tablet Enhancement of Cognition and Health (TECH) improves or preserves cognition in older adults with MCI.	MCI *n* = 61 IG: 30 CG: 31	IG: (75.6 [NR], 65–87) (F: *n* = 14, 46.7%; M: *n* = 16, 53.3%) CG: (75.1 [NR], 65–89) (F: *n* = 14, 45.2%; M: *n* = 17, 54.8%)	Community geriatric clinics (outpatient)	All used for T1, T2 and T3: 1. MOCA 2. WebNeuro 3. SF‐12	IG: TECH tablet self‐training 3–5×/week, 30–60 min for 5 weeks + weekly 60‐min OT group. CG: Standard OT for MCI single education session or 6 × 60‐min social game group (no home practice)	R: First and last authors I: IG TECH, weekly group participants; prescribed tablet apps; monitored logs/adherence; home self‐training; CG **s**tandard OT single education group or 6 × 60‐min social puzzle sessions. A: OT conducted all outcome assessments O: Nill	High attendance and good engagement with home practice in IG. Cognition (MoCA): IG improved significantly more than CG at T2 and T3; more IG participants achieved clinically meaningful gains.
Hassani Mehraban et al. 2024 *Iran* Q2 [51] *Interventional*	Compare International Classification of Functioning Core Set (ICF‐CS)–based occupational therapy with routine care in stroke patients.	Stroke *n* = 25 IG: 13 CG:12	IG: (54.92 [8.77], NR) CG: (56.83; [9.70], NR) IG: (F: *n* = 4, 16%; M: *n* = 9, 36%) CG: (F: *n* = 5, 20%; M: *n* = 7, 28%)	Private hospital (outpatient rehabilitation centre)	All used for T1, T2 and T3: 1. COPM 2. FM‐UE	IG: ICF‐Core Set–based OT (goal directed) CG: Routine OT including NDT Frequency (both groups): 3 × 45 min/week, 2 months	R: First and last authors I: CG provided routine OT including NDT. A: OT conducted all outcome measures O: IG unclear if they were study authors	IG: Improved more on FM‐UE scores from T1 to T2. COPM performance and satisfaction improved significantly in both groups from T1 to T2, with IG scoring significantly better than CG in both.
Levin et al. 2018 *Israel* Q1 (protocol study) [52] *Interventional*	Investigate personalised VR reaching training with/without anodal transcranial direct current stimulation (a‐tDCS) to improve UE function poststroke.	Stroke *n* = 60	NR [NR], 25–80) NR	Academic rehab centres (outpatient; Canada, Israel and India)	T1, T2 and T3: 1. MAS 2. FM‐UE 3. S‐WMFT	Group 1: Personalised VR reaching training (within active control zones) + anodal tDCS (a‐tDCS) over the affected hemisphere. Group 2: Nonpersonalised VR training (full elbow ROM) + a‐tDCS. Group 3: Personalised VR training + sham Frequency: 10 sessions/2 weeks, 50 min/day	R: Not OT I: OT delivering VR + tDCS A: NR O: Nill	N/A
Derakhshanfar et al. 2020 *Iran* Q1 [53] *Interventional*	To examine the impact of exteroceptive and proprioceptive sensory stimulation on upper limb motor function, reducing spasticity and improving activities of daily living in individuals with stroke.	Stroke *n* = 60 IG: 30 CG: 30	IG: (63.4 [10.85], 50–82) CG: (63.96 [9.37], 52–80) Total sample: (F: 23, 38%; M: 37, 62%)	Red Crescent Clinic (outpatient)	T1, T2 and T3: 1. BI 2. FM‐UE 3. MAS	IG: Conventional OT + sensory intervention (exteroceptive + proprioceptive stimulation per Rood principles: brushing 6 min, icing 5 min, stretch pressure 5 min, weight‐bearing 5 min side‐sitting + 5 min quadruped) CG: Conventional OT (ROM, strengthening and fine motor training). Frequency (both groups): 45 min/session, 4×/week, 6 weeks.	R: First author I: OT provided all intervention A: OT conducted all assessments O: Nill	IG: Improved more than CG on FM‐UE and BI from T1 to T2 (3 weeks) and T1 to T3 (6 weeks), with largest effects on FM‐UE. MAS scores improved more in IG than CG at T2 and T3. CG: Showed improvement over time (T1 to T3) on FM‐UE, BI and MAS, but gains were smaller than IG
Haji‐Ahmad et al. 2015 *Iran* No ranking [54] *Interventional*	To examine whether adding biofeedback therapy to routine occupational therapy improves hand function and ADLs in people after stroke.	Stroke *n* = 24 IG: 12 CG: 12	Total sample: (54.7 [2.6], NR). IG: (57.1 [2.9], NR) CG: (52.4 [4.3], NR) Total sample: F: 15 (62.5%); M: 9 (37.5%). IG: (F: *n* = 9, 75.0%; M: *n* = 3, 25.0%) CG: (F: *n* = 6, 50.0%; M: *n* = 6, 50.0%)	Rehab centre (outpatient)	T1 and T2: 1. BI 2. ROM (goniometer) 3. MAS	IG: Routine OT + surface biofeedback to wrist extensors (electrodes over wrist extensor bulk and lateral epicondyle; visual/audio feedback during targeted activation). CG: Conventional OT (stretching, positioning, facilitation/inhibition and tone normalisation). Frequency (both groups): 45 min/session, 3×/week, 3 months.	R: First author I: OT provided routine OT + biofeedback therapy A: Conducted all assessments O: Nill	IG: Improved more than CG from T1 to T2 on elbow, wrist and finger ROM, MAS and on the BI.
Saadatnia et al. 2020 *Iran* Q3 [55] *Interventional*	To examine whether a structured home‐based exercise programme improves ADLs and upper/lower limb function in acute ischemic stroke.	Stroke *n* = 40 IG: 20 CG: 20	IG: (62.0 [12.4], 33–80) CG: (66.0 [10.3], 40–80) Total sample: (F: 23, 57.5%; M: 17, 42.5%) IG: (F: 11, 55%; M: 9, 45%) CG: (F: 12, 60%; M: 8, 40%)	Education hospital (home based)	T1 and T2: 1. BI 2. FM‐UE + LE 3. MRS	IG: Structured 3‐month home programme (Month 1: Stretching/flexibility with caregiver; Month 2: Seated endurance + light resistance; Month 3: Balance + slow walking 1 h twice daily) + motivational training, monthly appraisal, weekly physiologist visit, motivational calls; routine PT/OT twice weekly in hospital. CG: Usual care only (no home programme or calls).	R: Not OT I: Provided sessions for both group at hospital + motivational training A: Not OT O: Nill	T1 to T2 significant difference, IG higher MRS than CG. IG better on BI, FM‐UE and FM‐LE. Within group T1 to T2 significant change in IG MRS and BI, FM‐UE and FM‐LE.
Rand et al. 2015 *Israel* Q1 (protocol study) [56] *Interventional*	Evaluate the effects of Rehab‐let (tablet‐based games) vs. GRASP on finger dexterity and satisfaction poststroke.	Stroke *n* = 40 IG: 20 CG: 20	(NR [NR], > 20) NR	Subacute rehab centre (inpatient)	T1 and T2: 1. 9‐HPT 2. Grip and pinch (Jamar dynamometer) 3. FM‐UE 4. ARAT 5. VAS Descriptive: 6. FIM	IG: Rehab‐let (touchscreen games) CG: GRASP protocol Frequency (both): 60 min/day, 5 ×/week, 4 weeks	R: First and last authors I: OT to guide and monitor progress A: NR O: Nill	N/A
Rand, et al. 2017 *Israel* Q1 [57] *Interventional*	Compare video‐game vs. traditional UE self‐training at home.	Stroke *n* = 24 IG: 13 CG: 11	IG: (59.1 [10.5], 33–75) CG: (64.9 [6.9], 58–80) IG: (F: *n* = 4, 30.8%; M: *n* = 9, 69.2%) CG: (F: *n* = 5, 45.5%; M: *n* = 6, 54.5%)	Rehab centre (home based)	All used for T1, T2 and T3: 1. ARAT 2. MAL 3. B&B 4. FRT Descriptive: 5. FIM	IG: Video‐game self‐training CG: Graded Repetitive Arm Supplementary Programme (GRASP) exercises. The traditional exercises included 15–25 exercises of seated activities such as arm stretches, cutting and pouring. Frequency (both groups): 1 h/day, 6×/week, 5 weeks.	R: First author I: Two OT home visits for each participant and remote guidance. A: Conducted assessments at T1, T2 and T3 O: Nill	The CG trained significantly longer than the IG despite similar functional gains. Both groups improved significantly in ARAT and MAL from T1 to T2, and improvements were maintained at T3. The IG showed higher follow‐up adherence.

Intervention: nonrandomised design (*n* = 8)								
Afshar, et al. 2024 *Iran* Q3 [58] *Interventional*	To describe and content‐validate a hand‐focused occupation‐based intervention (OBI) applied to two Iranian women with MS	MS *n* = 2	Participant 1: 41 years Participant 2: 50 years Both female	MS clinic (outpatient)	All used for T1 and T2: 1. COPM 2. 9‐HPT 3. FSS	IG: Hand‐focused OBI (MOHO‐based) 12 sessions (2×/week, 6 weeks); individualised, goal‐directed (one goal at a time) with coaching, practice, feedback, energy‐conservation and environmental strategies; progress tracked with COPM, 9HPT and FSS. CG: Not applicable.	R: First and last authors I: Co‐set meaningful goals, delivered a structured hand‐focused OBI, provided coaching/task adaptation/routine‐building, monitored progress. A: Screened MoCA, assessed COPM, 9HPT, FSS O: Reviewed the programme′s content validity.	The OBI content validity is relevant to be used in practice. Both achieved their self‐selected goals with meaningful gains on COPM and reduced FSS; 9HPT showed small improvements.
Alon et al. 2003 *Israel, Sweden and Netherlands* Q2 [59] *Interventional*	Test combined stimulation‐training programme for selected hand functions and impairments of chronic stroke survivors.	Stroke *n* = 77	(56.6 [NR], 44–67) NR	Rehab clinics (Israel, Sweden and the Netherlands) (clinic fitting and training of device + home programme)	All used for T1 and T2: 1. (J‐T) 2. B&B 3. 9‐HPT 4. MAS 5. VAS	IG: Handmaster hybrid orthosis and stimulation for 2 weeks. CG: Not applicable. Frequency: 3×/day home use + 1 clinic visit/week.	R: Not OT I: OTs fitted orthosis, provided training, done assessments A: Conducted all assessments with PT O: Nill	IG: Fine motor B&B scores improved, 9‐HPT time improved. VAS score reduced. MAS scores improved. 61.2% adhered to training. CG: N/A.
Berner et al. 2005 *Israel* Q2 [60] *Interventional*	To demonstrate the benefit of geriatric rehabilitation in West Nile fever (WNF) encephalitis patients.	Encephalitis‐WNF *n* = 5	(80 [NR], 77–85) (F: *n* = 4, 80%; M: *n* = 1, 20%)	Public hospital (inpatient)	All used for T1, T2 and T3: 1. MMSE 2. MEAMS 3. FIM	IG: MDT geriatric rehab (3–6 weeks avg. 35 ± 10 days). OT: ADL exercises + cognitive computer games. PT: Muscle strengthening and balance. CG: N/A.	R: Not OT I: OT delivered ADL and cognitive training A: NR O: Nill	IG: Improved FIM, regained continence, MMSE and MEAMS improved. CCT become normal. Gains maintained at 3 months. CG: N/A.
Mahmoudi Aqeel‐Abadi et al. 2022 *Iran* Q2 [61] *Interventional*	Evaluate structural and functional brain changes postmotor rehab in ischemic stroke.	Stroke *n* = 3	(60 [19.7], 38–75) (F: *n* = 1, 33.33%; M: *n* = 2, 66.66%)	Rehab centre (outpatient)	All used for T1 and T2: 1. FM‐UE 2. WMFT	IG: 4 weeks, 5×/week rehab combining OT (CIMT, BMT and PNF), strengthening, tDCS and NMES. CG: N/A.	R: Not OT I: OT delivered therapy such as PNF, CIMT and BMT. A: Conducted all assessments O: Nill	IG: Improved FM‐UE and WMFT scores in all patients, reduced iron, improved motor cortex activation and improved corticospinal integrity. CG: N/A.
Soufi Ahmadi et al. 2019 *Iran* Q3 [62] *Interventional*	To examine the effect of 12 sessions of traditional occupational therapy (TOT) vs, TOT + VR (E‐Link) for upper limb function after stroke.	Stroke *n* = 30 IG: 15 CG: 15	IG: (55.23 [NR], 36–72) CG: (55.26 [NR], 39–80) IG: (F: 4, 26.66%; M: 11, 73.33%) CG: (F: 6, 40.0%; M: 9, 60.0%).	OT laboratory (outpatient)	T1 and T2: 1. FM‐UE 2. CAHAI 3. SIS 4. MAS 5. Motricity Index 6. ROM (goniometer)	IG: 40 min VR (E‐Link) + traditional OT (distal UE focus; graded task‐like games for wrist/forearm). CG: 60 min Traditional OT (strengthening, ROM, tabletop/ADL practice) matched to IG content. Frequency: 4 weeks; 3×/week, 60 min.	R: First author I: OT provided all intervention A: OT conducted all assessments O: Nill	IG did better than CG FM‐UE total and wrist/hand subparts, strength Motricity Index and CAHAI. IG also showed larger gains in forearm supination and wrist extension ROM and greater reductions MAS. No between‐group difference was found on SIS at post‐test.
Mehdizadeh et al. 2017 *Iran* Q3 [63] *Interventional*	Evaluate effect of group‐based OT on ADL performance and ADL satisfaction for stroke survivors.	Stroke *n* = 14 IG: 7 CG: 7	Total sample: (52 [10.6], NR). IG: (49 [9.66], NR). CG: (55 [10.1], NR) IG: (F: NR; M: 3, 42.8%) CG: (F: NR; M: 6, 85.7%)	ADL clinic (outpatient)	T1 and T2: 1. BI 2. COPM 3. MRS	IG: Circuit class therapy (neuro‐occupation); emphasis on affected‐side use, adaptive equipment and peer interaction. Structure: 20‐min UE/LE mobility, 40‐min craft tasks (e.g., photo frame, colouring, ceramics and sculpting), 40‐min cooking/eating tasks (cutting, peeling, cooking, utensil handling and independent eating). CG: Usual one‐on‐one outpatient OT. Frequency: six sessions, 120 min each	R: First author I: Provided intervention A: Conducted assessments at T1 O: A 20‐min part of the session was unspecified.	Both groups improved from T1 to T2. IG showed greater gains than CG on COPM performance and COPM satisfaction (time × group). At post‐test, only COPM satisfaction was higher in IG than CG.
Ostrei et al. 2020 *Israel* Q3 [64] *Interventional*	Assess the effect of Feuerstein Instrumental Enrichment (FIE) for cognitive and functional change in elderly MCI patients.	MCI *n* = 9	(76.2 [4.9], 67–81) (F: *n* = 4, 44.4%; M: *n* = 5, 55.6%)	Geriatric assessment unit (outpatient)	All used for T1, T2 and T3: 1. MoCA—Hebrew version 2. NeuroTrax (Mindstream) 3. ACS 4. COPM 5. DEX	IG: 30 FIE sessions (2×/week, 90 min), led by Feuerstein teacher. OT conducted baseline/follow‐up assessments and qualitative feedback. CG: N/A.	R: First author I: OT delivered assessments, follow‐ups, coordinated with FIE sessions A: Conducted all assessments at T1, T2 and T3 O: Nill	IG: Improved visuospatial subtest of NeuroTrax (Mindstream), MoCA, and sustained gains at T3. Participants reported cognitive, emotional and social benefits. CG: N/A.
Weiss et al. 2003 *Israel* Q3 [65] *Interventional*	Evaluate feasibility of desktop VR for training street crossing in stroke with spatial neglect.	Stroke with (unilateral spatial neglect) *n* = 12	(NR [NR], 55–75) Not specified	NR (simulated VR city environment)	None	IG: Desktop VR street‐crossing simulation. Included progressive difficulty over 12 sessions (30 min each) within 4 weeks. Training incorporated real‐time OT feedback. CG: Age‐matched controls (for comparison only) Frequency: 3×/week, 4 weeks (total 12 sessions, 30 min each).	R: First and last authors I: OTs used an off‐the‐shelf 3D website development authoring tool to develop a simulated VR training intervention for safely crossing the street A: Nill O: OTs contributed to development of VR scenarios	IG and CG participants adapted to the VR street‐crossing task and found it engaging. CG crossed faster, with fewer accidents and fewer attempts, whereas IG crossed more slowly, had more accidents and checked traffic more frequently. All IG and CG participants experienced accidents, though fewer occurred in CG.

Intervention: cohort design (prospective and retrospective) *n* = 3								
Chowdhury and Leenen 2021 *Saudi Arabia* Q1 [66] *Interventional*	Compare the outcomes regarding discharge destination and length of hospital stay in traumatic brain injury patients before and after launching an acute intensive trauma rehabilitation (AITR) programme.	Traumatic brain injury Pre‐AITR group: *n* = 108 Post‐AITR group: *n* = 111	Pre‐AITR: (26.9 [14.1], NR) (F: *n* = 8, 7.4%; M: *n* = 100, 92.6%) Post‐AITR: (29.4 [14.3], NR) (F: *n* = 16, 14.1%; M: *n* = 95, 85.5%)	Public hospital (inpatient and Physical Medicine and Rehabilitation Department)	All used for T1 and T2: 1. GCS 2. ISS 3. AIS‐head	Pre‐AITR is cohort who had nonintensive rehab before the programme was introduced AITR: Structured MDT programme (Dec. 2018–Dec. 2019): two to three sessions/day × 3–4 h, 5 days/week. Included OT, PT, ST, rehab medicine, orthotics/prosthetics, social work and botox therapy as needed. Compared to nonintensive rehab (Aug. 2017–Nov. 2018).	R: Not OT I: OT delivered functional retraining (self‐care and instrumental ADL). A: NR O: Nill	AITR group had significantly shorter hospital stay. Discharge to home significantly higher in AITR patients vs. discharge to institutional settings. Patients with AITR had more severe injuries than pre‐AITR group but still made greater improvement.
Keren et al. 2004 *Israel* Q2 [67] *Interventional*	Evaluate the relationship between therapy intensity (especially OT) and outcomes in stroke rehab.	Stroke *n* = 50	(Media*n* = 63 [NR], 39–83) (F: *n* = NR, NR; M: *n* = NR, 64%)	Rehab hospital (inpatient)	All used for T1 and T2: 1. MMSE 2. FIM 3. BI 4. Stroke Impairment Assessment Set 5. NIHSS 6. RIC‐FAS	Routine inpatient stroke rehabilitation programme. Therapy intensity logged for OT, PT and SLP.	R: Not OT I: Delivered OT focusing on ADLs, IADLs, cognition, mobility and community integration A: Conducted nine items from FIM and RIC‐FAS O: Nill	Seventy‐eight percent discharged home, 14% to other care. Significant FIM gains. Higher OT intensity linked to motor/cognitive improvement. Younger age, better T1 function and longer rehab predicted better outcomes.
Weiss et al. 2004 *Israel* Q2 [68] *Interventional*	Compare effectiveness and cost‐efficiency of home‐based vs. inpatient OT rehabilitation poststroke.	Stroke *n* = 191 Home rehabilitation group (HR) = 98 Institutional rehabilitation group (IR) = 93	IR: (67.6 [NR], 39–89) (F: *n* = NR, 41.8%; M: *n* = NR, 63.4%) HR: (69.7 [NR], 28–92) (F: *n* = NR, 58.2%; M: *n* = NR, 36.6%)	Rehab institutions and patients′ homes (inpatient and home‐based rehab)	All used for T1 and T2: 1. BI 2. FAI	HR: Average 5 OT visits (45–60 min), 1/week over 57 days. OT did ADL exercises, trained patients and family. PT: 14 visits. MDT also included nurse and physician. Total cost $442/patient. IR: Inpatient rehab at four facilities. Four to five OT/PT sessions/week (45 min), avg. 60 days. Cost $320/day.	R: Not OT I: Delivered home‐based and inpatient OT sessions A: NR O: Nill	HR: Significant improvement in BI (ADL/mobility areas). Cost‐effectiveness: significantly cheaper. Regression: BI scores = stronger predictor of FAI than rehab type.

Other designs *n* = 3								
Asly and Hazim 2020 *Morocco* Q3 [69] *Interventional*	Describe Morocco′s health situation during the 2019 COVID pandemic and develop prerehabilitation guidelines for COVID‐19 patients who continue to experience disabilities.	Post–COVID‐19 (including neurological sequelae) Not specified	NR NR	Acute care hospital (inpatient, outpatient, ambulatory and home care)	None	MDT including doctors, physio, OT, nurses; neuromotor rehab (passive mobilisation, strengthening, functional exercises and verticalisation); neuropsych rehab (speech therapy, OT, psychological care and reconditioning) offered after cognitive assessment.	R: Not OT I: OT focused on elderly ADL difficulties, facilitating return home. A: N/A O: Nill	MDT and pharmacological/nonpharmacological interventions offered in various care settings post‐COVID.
Darvishi et al. 2023 *Iran* Q2 [70] *Interventional*	Evaluate the cost‐effectiveness of MDT rehabilitation for stroke survivors in Iran by comparing outcomes and costs between patients receiving MDT rehab and those without rehab.	Stroke *N* = 100,000 stroke patients/year.	(67 [NR], NR) NR	Public and private hospitals; multidisciplinary stroke rehabilitation services (NR)	T1 and T2: 1. BI	Comparison of patients receiving MDT rehabilitation including doctors, PTs, OTs and nurses; initiated postclinical stabilisation for 6 months vs. no rehabilitation. No detailed rehab process provided; based on benchmark studies.	R: Not OT I: OTs were part of a MDT providing rehabilitation services, contributing to functional recovery and QoL improvements. A: NR O: Nill	Using public tariffs, the rehabilitation strategy was dominant lower cost (US$5320 vs. US$6047) and higher QALYs (2.78 vs. 2.61) and cost‐effective in 96.6% of Monte Carlo simulations. Under private tariffs, it was cost‐effective in only 55.2% of iterations, with less clear economic advantage. Rehab saved US$4276 per QALY gained vs. no rehab when public tariffs were applied.
Katz and Hadas 1995 *Israel* Q1 [71] *Interventional*	Present occupational therapy cognitive models used in psychiatric rehabilitation, focusing on both remedial and functional approaches.	Psychiatric cases *n* = 2 (case examples)	Case 1: 27 years Case 2: 32 years Case 1: (Sex not specified) Case 2: Male	Psychiatric hospital (inpatient)	Descriptive: 1. ACLS 2. CPT 3. RTI	Functional approach (Case 1): Structured, graded OT tasks (e.g., bookbinding); Remedial approach (Case 2): Five stages of cognitive training using IE programme.	R: First and last authors I: Functional approach: OT matched tasks to cognitive level, structured learning for work placement. Remedial: OT used Instrumental Enrichment (IE) to rebuild cognitive and emotional resilience. A: OT conducted ACLS O: Nill	OT used cognitive rehab models to structure interventions. Case 1 showed gains in structured ADLs and work readiness; Case 2 showed improved confidence and reduced anxiety. Emphasised importance of tailored approaches in psychiatric OT.

*^a^Eligibility and nonstandardised assessments excluded.*

*^b^Researcher role is only included for the first and/or last author.*

*^c^Only significant outcomes according to authors are reported*; citation details for all assessments are in File S1.

Abbreviations: ACLS, Allen Cognitive Level Screen; ACS, Activity Card Sort; ADLs, Activities of Daily Livings; AIS‐head, Abbreviated Injury Scale (head); ARAT, Action Research Arm Test; AROM, active range of motion; B&B, Box and Block Test; BI, Barthel Index; BMT, Brunnstrom Movement Therapy; CAHAI, The Chedoke Arm and Hand Activity Inventory; CIMT, Constraint‐Induced Movement Therapy; COPM, Canadian Occupational Performance Measure; CPT, Cognitive Performance Test; CTT, Colour Trails Test; DEX, Dysexecutive Questionnaire; EFs, executive functions; FAI, Frenchay Activities Index; FIM, Functional Independence Measure; FM‐UE, Fugl–Meyer Assessment of the Upper Extremity; FM, The Brunnström–Fugl–Meyer test; FRT, Functional Reach Test; FSS, Fatigue Severity Scale; GCS, Glasgow Coma Scale; GRASP, Graded Repetitive Arm Supplementary Programme; I‐PEX, Intervention of Participation and Executive Functions; IADL, Instrumental Activities of Daily Living; ISS, Injury Severity Score; J–T test, Jebsen–Taylor Hand Function Test; LE, lower extremity; LOTCA, Loewenstein Occupational Therapy Cognitive Assessment; MAL, Motor Activity Log; MAS, Modified Ashworth Scale; MCI, mild cognitive impairment; MDT, multidisciplinary team; MEAMS, Middlesex Elderly Assessment of Mental State; MET‐HV, Multiple Errands Test, Hospital Version; MMSE, Mini‐Mental State Examination; MOHO, Model of Human Occupation; MoCA, Montreal Cognitive Assessment; MPAI‐4, Mayo‐Portland Adaptability Inventory‐4; MRS, Modified Rankin Scale; MS, multiple sclerosis; N/A, not applicable; NR, not reported; NDT, neuro‐developmental treatment; NIHSS, National Institutes of Health Stroke Scale; NMES, Neuromuscular Electrical Stimulation; OT, occupational therapy; 9‐HPT, Nine‐Hole Peg Test; OTs, occupational therapists; PNF, proprioceptive neuromuscular facilitation; PT, physical therapy; QoL, Quality of Life; QOLIBRI, Quality of Life after Brain Injury; ROM (goniometer), range of motion; RCTs, randomised controlled trials; RIC‐FAS, Rehabilitation Institute of Chicago Functional Assessment Scale; RTI, Routine Task Inventory; SF‐12, 12‐Item Short Form Health Survey; SIS, Stroke Impact Scale; SLP, speech language pathologist; SPIRQ, Self‐Perceptions in Rehabilitation Questionnaire; S‐WMFT, Streamlined Wolf Motor Function Test; TECH, Tablet Enhancement of Cognition and Health; UE, upper extremity; VAS, Visual Analogue Scale; VR, virtual reality; WMFT, Wolf Motor Function Test.

**Table 4:** Nonintervention observational (cohort, cross‐sectional, multimethod and other designs).

Author Year *Country* Journal ranking Question type *Aim 1* *Aim 2*	Aim	Condition Sample size (*n*) Intervention group (IG) Control group (CG)	Age (years) (mean [SD], range) Sex (F [*n*], %; M [*n*], %)	Context (setting)	^a^Standardised assessments used Outcome measures across time: T1: (Baseline) T2: (Postintervention) T3: (Follow‐up) Or descriptive measure	Intervention overview	OT role(s) ^c^R (researcher) I (interventionist) A (assessor) O (other)	^b^Outcome
Cohort design (prospective, retrospective and longitudinal) *n* = 8								
Asirvatham et al. 2022 *Qatar* Q4 [72] *Prognostic*	To describe functional gain in physical performance and well‐being and identify predictors of functional gain in post‐COVID stroke survivors receiving active rehab.	Stroke (post‐COVID) *n* = 20	(56 [NR], NR) (M: *n* = 20, 100%.)	Public hospital (inpatient)	All used for T1 and T2: 1. MMSE 2. FIM 3. PCFS 4. FAC 5. Borg RPE 6. ARAT 7. NIHSS	Interdisciplinary active rehab provided; length of stay reported; no further programme details given.	R: First author I: NR A: NR O: Nill	All the following had significant improvements across all outcomes PCFS, MMSE, ARAT, Borg RPE, FAC and NIHSS. Regression not reported; only bivariate correlations presented.
Greenberg et al. 2006 *Israel* Q2 [73] *Prognostic*	Examine functional improvement and outcome in patients with primary brain tumours and compare them to stroke patients.	Stroke and brain tumours (glioma and meningioma) Stroke: *n* = 1660 Meningioma: *n* = 128 Glioma: *n* = 40	Stroke: (60.4 [14.5], NR) (F: *n* = NR, 30%; M: *n* = NR, 70%) Meningioma: Mean: (59.9 [15.6], NR) (F: *n* = NR, 38%; M: *n* = NR, 62%) Glioma: Mean: (54.1 [13.4], NR) (F: *n* = NR, 50%; M: *n* = NR, 50%)	Public hospital (inpatient)	Used for T1 and T2: 1. FIM Descriptive: 2. LOTCA 3. ILAT	All groups received MDT inpatient rehab. ILAT used for communication. PT assessed tone, balance and mobility. Stays were shorter for tumour groups; stroke patients admitted sooner post‐onset.	R: Not OT I: NR A: OT conducted ADL assessment within 3 days of admission using LOTCA O: Nill	All groups improved function with MDT rehab. Meningioma patients showed higher function at T2 than stroke or glioma. Despite shorter stays, both tumour groups improved. Stroke admissions shorter than in previous years. Functional gains were achievable even with brief rehab prior to community discharge or further treatment.
Loni et al. 2024 *Iran* Q3 [74] *Prognostic*	Aim 1: Assess functional changes using FIM and SCIM III from baseline to post‐inpatient rehabilitation. Aim 2: Compare improvements across injury levels and severities. Aim 3: Examine prognostic factors influencing functional independence in patients with SCI.	SCI *n* = 180	(34.47 [13.2], 16–73) (F: *n* = 51, 28%; M: *n* = 129, 72%)	Rehab hospital (inpatient)	All used for T1 and T2: 1. FIM 2. SCIM III	The study mentioned need for MDT in background section, but no description of rehab programme or team given.	R: Not OT I: NR A: OT measured FIM and SCIM III at T1 and T2. O: Nill	Patients showed significant functional improvements at T2: Aim 1 and 2: SCIM III and FIM had greatest gains in patients with lower level injuries (e.g., T10–L2). Aim 3: Pressure ulcers significantly reduced outcomes, especially at higher grades. Lower injury levels and longer hospital stays were linked to better functional gains.
Mahmoud et al. 2017 *Saudi Arabia* No ranking [75] *Prognostic*	Examine rehabilitation timelines and outcomes and identify predictors of motor recovery in patients with SCI.	SCI *n* = 418 Traumatic SCI: *n* = 312 Nontraumatic SCI: *n* = 106	Traumatic SCI: (31.4 [NR], NR) (F: *n* = 68, 22%; M: *n* = 244,78%.) Nontraumatic SCI: (43.8, [NR], NR) (F: *n* = 28, 26%; M: *n* = 78, 74%.)	Rehab hospital (inpatient and outpatient)	Used for T1 and T2: 1. SCIM‐III Descriptive: 2. ASIA‐AIS	General description about the daily multidisciplinary rehab 5–6 h/day × 5 days/week. OT: 1 h/day, PT: 2–3 h/day. Group, art and recreational therapy also included.	R: Last author I: NR A: NR O: Nill	Significant FIM motor score improvements were seen in both groups. T1 FIM score predicted T2 FIM score. Traumatic SCI: Tetraplegia + delayed admission led to poorer outcomes. Nontraumatic SCI: Older age + complete and sensory incomplete injuries led to worse outcomes.
Qannam et al. 2017 *Saudi Arabia* Q2 [76] *Prognostic*	To explore correlates of admission pathway, length of stay, functional outcome and TBI patient attributes.	Traumatic brain injury *n* = 208	F: 28 [NR], 16–98) M: (31 [NR], 17–77) (F: *n* = 36, 17%; M: *n* = 172, 83%)	Public hospital (inpatient)	Used for T1 and T2: 1. FIM	Observational study using retrospective chart review (2009–2014). FIM administered at T1 and T2 to assess function.	R: First and last authors I: N/A A: NR O: Nill	Significant FIM improvements were found for all groups, with the acute care group achieving greater total and cognitive gains exceeding the Minimal Clinically Important Difference (MCID). FIM admission score was the strongest predictor of discharge outcome; delayed rehabilitation admission had a small negative effect, whereas length of stay was not a significant contributor.
Rayegani et al. 2016 *Iran* Q3 [77] *Prognostic*	Evaluate functional status using the FIM at admission, discharge and 6‐month poststroke, and to examine functional changes over time.	Stroke *n* = 108	(62 [17], 16–91) (F: *n* = 38, 35%; M: *n* = 70, 65%)	Public hospital (inpatient and outpatient)	Used for T1, T2 and T3: 1. FIM	Routine care (inpatient and outpatient follow‐up; no intervention specified)	R: Not OT I: NR A: Not OT O: Nill	Higher T1 FIM scores predicted better at T2 and T3. Motor independence linked to better cognitive scores. Age negatively impacted functional outcomes at T2 and T3 but not at T1. Lower initial FIM scores correlated with high improvements. Longer hospital stays associated with lower FIM scores at T2 and T3.
Yekaninejad. et al. 2024 *Iran* Q1 [78] *Prognostic*	Assess changes in Spinal Cord Independence Measure (SCIM‐III) scores over time and examine the influence of various factors on functional improvement.	SCI *n* = 559	Total sample: (42.06 [10.6], 18–60) Age subgroups: *n* = 54 (9.7%), 18–30 *n* = 464 (83%), 31–60 (F: *n* = 112, 20%; M: *n* = 447, 80%)	Brain and spinal injury research centre (outpatient)	Used for T1, T2 and T3: 1. SCIM‐III Descriptive: 2. ASIA‐AIS	6‐month structured rehabilitation programme. Face‐to‐face education + group discussion. 3 h every 2 months, delivered by a multidisciplinary team. SCIM‐III administered at multiple points over 8 years.	R: Not OT I: Delivered face‐to‐face education; 2‐h sessions, 2×/week. A: NR O: OT participated in group sessions	SCIM‐III increased from across groups. Predictors of better outcome include younger age, married, university educated, employed and lower level injury (e.g., lower thoracic) injuries.
Zwecker et al. 2002 *Israel* Q1 [79] *Diagnostic*	Aim 1: Compare 3 cognitive tools (LOTCA, MMSE, FIM cognitive subscale). Aim 2: Assess their ability to predict functional outcomes at discharge.	Stroke *n* = 66	(72 [8.9], 47–87) (F: *n* = 17, 26%, M: *n* = 49, 74%)	Geriatric neurologic rehab department (inpatient)	All used for T1 and T2: 1. MMSE 2. LOTCA 3. FIM (+ cognitive subscale)	Routine inpatient rehab. Cognitive assessments performed by OT at T1 and T1.	R: Not OT I: N/A A: Conducted all assessments O: Nill	Aim 1: Significant improvement in total FIM motor, cognitive. LOTCA score increased whereas MMSE only at T1. Aim 2: Correlation: There were moderate positive correlations observed between MMSE, LOTCA and FIM‐cognitive scores. LOTCA best predictor of FIM motor. All three tools weak in predicting efficiency.

Cross‐sectional design *n* = 15								
Abu Tariah et al. 2020 *Saudi Arabia* Q2 [80] *Prognostic*	Aim 1: Assess achievement of OT goals for stroke rehabilitation patients in Saudi Arabia. Aim 2: Examine relationship between goal achievement and patient characteristics. Aim 3: Evaluate quality of written OT goals.	Stroke *n* = 100	(59 [NR], 23–87) (F: *n* = 38, 38%; M: *n* = 62, 62%)	Public hospital (inpatient and outpatient)	Descriptive: 1. GAS	N/A	R: First author I: OT responsible for goal setting with patients; evaluation based on goal achievement using GAS. A: NR O: In GAS score and type of goal, assumption is that this was done either by the treating OT (if prospective) or the research team (if retrospective).	Aim1: Total goals = 249. Fifty‐four percent achieved. Most were performance skills 52%; only 30% were functional. Aim 2: No differences by age, gender, type/side of stroke. Aim 3: Only 30% of goals had all SMART criteria.
Almosallam et al. 2022 *Saudi Arabia* Q1 [81] *Prognostic*	Examine the contributing factors influencing return to driving among stroke survivors within the Saudi population.	Stroke *n* = 100	*n* = 9 (9%), 18–30; *n* = 31 (31%), 31–50; *n* = 60 (60%), ≥ 51 (Only M: *n* = 100, 100%)	Stroke rehabilitation clinics (outpatient)	None	N/A	R: Not OT I: N/A A: N/A O: OT conducted all interviews via telephone.	Ninety‐four percent drove prestroke; only 7% returned to driving. Main reasons for not driving: poor health, lack of confidence, lack of support. No demographic associations.
Alqahtani 2015 *Saudi Arabia* Q4 [82] *Diagnostic*	To evaluate the clinical utility of a test battery for detecting spatial neglect in Arabic‐speaking stroke patients, considering the unique right‐to‐left orientation of reading and writing in Arabic compared to Latin‐based languages.	Stroke *n* = 165	(54 [NR], 34–71) (F: *n* = 49, 29.7%; M: *n* = est.116)	Public hospital (inpatient)	Descriptive: 1. The clock drawing test 5. The bell cancelation test	N/A	R: Not OT I: N/A A: NR O: OT mentioned as part of MDT	Bell cancellation test detected highest prevalence (41.2%); clock drawing and line bisection had lowest detection. No single test detected all cases.
Bar‐Yosef et al. 2000 *Israel* Q4 [83] *Diagnostic*	Investigate reliability (including inter‐rater agreement and internal consistency) and validity (both construct and concurrent) of the CPT among elderly Israelis, both with and without dementia.	Dementia *n* = 60 Dementia group: *n* = 30 CG: *n* = 30	Dementia: (82 [7.27], 65–97) CG: (81 [6.11], 68–92) Both groups included: (F: *n* = 17, 56.6%, M: *n* = 13, 43.3%)	Community‐based clinics and dementia care facilities (outpatient)	Descriptive: 1. CPT 2. RTI‐II	N/A	R: First and last authors I: N/A A: Conducted all assessments O: OTs translated/adapted CPT; Conducted inter‐rater reliability scoring from video	CPT showed excellent inter‐rater reliability, high internal consistency, strong concurrent and construct validity. Discriminated well between dementia and CG.
Cermak et al. 1995 *Israel* Q1 [84] *Diagnostic*	Examine cross‐cultural differences in LOTCA scores between American and Israeli stroke patients and compare cognitive profiles between individuals with right versus left cerebral vascular accidents, given the lack of separate normative data for these subgroups.	Stroke (RCVA/LCVA) *n* = 81 United States: *n* = 25 Israel: *n* = 56	United States: RCVA: (64.3 [10.2], 40–80) – LCVA: (69.0 [13.6], 40–80) Israel: – RCVA: (58.5 [7.1], 40–80) – LCVA: (55.0 [9.4], 40–80) Total sample (*n* = 81): (F: *n* = 24 29.6%; M: *n* = 57 70.4%) RCVA (*n* = 45): (F: *n* = 13 28.9%; M: *n* = 32 71.1%) LCVA (*n* = 36): (F: *n* = 11 30.6%; M: *n* = 25 69.4%)	‐ United States: Rehab hospital and long‐term care facility ‐ Israel: Rehab hospital (inpatient)	Descriptive: 1. LOTCA	N/A	R: First and last authors. I: N/A A: LOTCA conducted by clinical OT in the United States. In Israel, administered by the fourth author. O: Nill	Israeli participants scored higher than Americans on several LOTCA subtests, particularly in orientation to time, drawing a clock, object classification with (RCVA) and object constancy with (LCVA). RCVA participants outperformed LCVA on some subtests in both American and Israeli samples, whereas LCVA performed better on pegboard construction across both groups. LOTCA showed limited ability to discriminate between RCVA and LCVA.
D′Cunha et al. 2025 *Israel* Q1 [85] *Diagnostic*	Investigate content validity of Version 6 of the Handwriting Assessment Battery (HAB‐v6) for handwriting post acquired brain injury	Stroke and traumatic brain injury No patients; *n* = 15 therapists	NR NR	Online survey across the United States, the United Kingdom, Canada, Israel and Australia. (Not applicable)	Descriptive: 1. HAB‐v6	N/A	R: First and last authors I: N/A A: N/A O: 11 expert OTs in handwriting assessment participated in Delphi rounds	More than 70% agreement on 5/6 subtests′ importance; consensus on adding/modifying tasks. Poor consensus on dot subtest. Study supports content validity refinement.
Dehghan et al. 2019 *Iran* Q4 [86] *Prevalence*	Identify occupations perceived as difficult by individuals with MS across self‐care, productivity and leisure domains, and to examine the relationship between disability level and mean performance and satisfaction scores among 20–50‐year‐old individuals with MS attending rehabilitation centres in Arak, Iran.	MS *n* = 50	(38.2 [7.40], 20–50) (F: *n* = 43, 86%; M: *n* = 7, 14%)	Iranian MS society referrals and rehab centres (Not specified)	Descriptive: 1. COPM—Persian version 2. EDSS	N/A	R: First author I: N/A A: OT administered COPM to assess self‐care, productivity and leisure priorities. O: Nill	People with mild to moderate MS most often struggled with self‐care and household management tasks. Of 248 difficult occupations, household management was the most common and lowest performing. Prioritised tasks showed moderate performance and low satisfaction. Greater MS severity was linked to lower performance.
Ghaffari et al. 2021 *Iran* Q2 [87] *Prevalence*	To determine factors predicting IADL in stroke patients	Stroke *n* = 90	(NR [NR], 30–80) (F: 43, 52.4%; M: 39, 47.6%)	OT clinic NR	Descriptive: 1. WMS‐R 2. TMT 3. Lawton IADL scale 4. BI 5. Motricity Index 6. BDI‐II	N/A	R: First and last authors I: N/A A: OT conducted all assessment O: Nill	Regression identified BADL as the main predictor of IADL; better TMT performance was associated with higher IADL, whereas older age and greater depression were associated with poorer IADL; other factors measured in the study were not present in the explanatory model.
Fallahpour et al. 2011 *Iran* Q2 [88] *Diagnostic*	Assess psychometric characteristics of the Persian Impact on Participation and Autonomy (IPA‐P) for stroke patients.	Stroke *n* = 102	(58.3 [11.9], 27–75) (F: *n* = 42, 41.2%; M: *n* = 60, 58.8%)	Two university hospitals and two university rehab centres (outpatient)	Descriptive: 1. BI 2. FM‐UE 3. IPA‐P	N/A	R: First and last authors I: N/A A: OT conducted all assessments O: OT conducted all interviews	Rasch model supported two constructs in IPA‐P: (1) performance‐based and (2) social‐based participation. Provided evidence of internal validity, person‐response validity and sensitivity for both.
Fallahpour et al. 2011 *Iran* Q1 [89] *Prevalence*	Explore perceived participation and autonomy in a group of stroke survivors in Iran and identify factors that influence poststroke participation.	Stroke *n* = 102	(58.3 [11.9], 27–75) (F: *n* = 42, 41.2%; M: *n* = 60, 58.8%)	University rehab clinics (outpatient)	Descriptive: 1. MMSE 2. BI 3. FM‐UE 4. IPA‐P 5. HADS 6. SIS‐16	N/A	R: First and last authors I: N/A A: OTs conducted all assessments O: OTs conducted interviews.	Most reported participation and autonomy as good–fair except in outdoor autonomy. Physical function predicted performance‐based participation; mood predicted social‐based participation.
Hamed and Holm 2013 *Jordan* Q3 [90] *Diagnostic*	Examine psychometric properties of the newly developed Arabic Heritage Activity Card Sort (A‐ACS) in Jordanian adults, focusing on concurrent, discriminative and convergent validity. Internal consistency and test–retest reliability	MS *n* = 105 MS: *n* = 43 Healthy: *n* = 62	Total sample: (45.1 [17.1], 72% 20–59) MS: (40.8 [9.9], NR) Healthy: (48.0 [20.2], NR) (F: *n* = 57, 54.3%; M: *n* = 48, 45.7%)	MS society and local community gatherings (MS society building or participant′s house)	Descriptive: 1. A‐PASS‐SR 2. A‐ACS 3. A‐MPAI‐4	N/A	R: First and last authors I: N/A A: OT conducted all assessments O: OTs retested A‐ACS	A‐ACS showed good concurrent, discriminative and convergent validity. Internal consistency high. Strong psychometrics support use in Arabic‐speaking populations.
Hamed et al. 2012 *Jordan* Q2 [91] *Diagnostic*	Translate the MPAI‐4 into Arabic and evaluate the psychometric properties of the Arabic version (A‐MPAI‐4) among healthy adults as well as patients diagnosed with MS and stroke.	Stroke and MS *n* = 128 Stroke: *n* = 17 MS: *n* = 49 Healthy: *n* = 62	Total sample: (45.68 [17.57], 18–80) MS: (39.37 [1.47]) Stroke: (54.29 [3.91]) Healthy: (48.31 [2.63]) (F: *n* = 72, 56.25%; M: *n* = 56, 43.75%)	Public hospital, royal medical services hospital; local society (outpatient)	Descriptive: 1. A‐PASS‐SR 2. A‐MPAI‐4	N/A	R: First and last authors I: N/A A: OT conducted all assessments O: OT led forward–backward translation, finalised Arabic MPAI‐4 and conducted test–retest assessment sessions.	The A‐MPAI‐4 showed strong discriminative validity between stroke and MS groups vs. healthy controls. Post hoc had significant differences between both patient groups and the healthy group. It showed moderate convergent validity with the A‐PASS‐SR and strong test–retest reliability.
Kirshenboim, et al. 2025 *Israel* Q1 [92] *Prevalence*	To validate the DO‐IT test which assesses dual‐task capacity involving the upper extremity. Validation with stroke survivors and healthy controls in younger and older age groups.	Stroke *n* = 115 Stroke: 83 Healthy: 32	Stroke: Median 65 (IQR; 53–72); range: (22–87) younger group (20–54); older group (55–90) Healthy: Younger group (22–54); older group (56–87) Stroke: (F: *n* = 38 45.7%; M: *n* = 45 54.3%) Healthy: F: *n* = 21 65.6%; M: *n* = 11 34.4%)	Hospital and community (inpatient and outpatient)	Descriptive: 1. MoCA 2. CTT	N/A	R: First and last authors I: N/A A: OTs conducted all assessments O: nill	Younger > older on dual‐task motor and walking; stroke < healthy on MoCA and DO‐IT; in healthy, DO‐IT correlated with walking dual‐task; in stroke, DO‐IT correlated with MoCA/CTT.
Malkawi et al. 2024 *Jordan* Q4 [93] *Prevenance*	Examine how stroke affects QoL and the impact of demographic/clinical factors	Stroke *n* = 64	(58.9 [12.4], 20–80) (F: *n* = 24, 37%; M: *n* = 40, 63%)	Three major rehabilitation hospitals (inpatient and outpatient)	Descriptive: 1. SIS	N/A	R: First author I: N/A A: OT conducted SIS O: OT conducted face‐to‐face interviews using SIS.	QoL moderate; lowest in hand function, highest in communication. QoL affected by education, stroke type, history and aphasia. Aphasia impacted communication and memory domains.
Naghdi et al. 2016 *Iran* Q1 [94] *Diagnostic*	Translate, culturally adapt and validate Persian FIM for stroke patients in Iran.	Stroke *n* = 40	(60 [14.9], 22–82) (F: *n* = 15, 37.5%; M: *n* = 25, 62.5%)	Tabassum centre of stroke rehabilitation (outpatient)	Descriptive: 1. BI‐Persion version 2. PFIM	N/A	R: Not OT I: N/A A: OT conducted all assessments O: OTs translated and adapted FIM into Persian (PFIM), ran reliability testing and led expert review on PFIMs use in Persian‐speaking populations.	Results found high reliability and internal consistency, strong PFIM‐PBI correlation, three‐factor model (mobility, sphincter and cognition). Valid and culturally appropriate for Persian speakers.

Mutimethod design *n* = 4								
Al‐Haidary et al. 2015 *Saudi Arabia* Q1 [95] *Diagnostic*	Develop and evaluate a goal‐setting tool (goal menu) for neurological rehabilitation.	Stroke, traumatic brain injury, SCI and other neurological disorders. *n* = 130 Stroke: *n* = 31 Traumatic brain injury: *n* = 19 SCI: *n* = 46 Other neurological disorders: *n* = 34 Health professionals: Group 1: *n* = 9 Group 2: *n* = 21	All patients: 19–30 (*n* = 59, 45.4%) 31–40 (*n* = 14, 10.8%) 41–50 (*n* = 14, 10.8%) 51–60 (*n* = 15, 11.5%) ≥ 61 (*n* = 18, 13.8%) 13–18 (*n* = 10, 7.7%) (F: *n* = 38, 29.2%; M: *n* = 92, 70.8%) Health professionals: NR	Rehab hospital (inpatient)	Descriptive: 1. FIM 2. COPM 3. Rivermead Life Goals Questionnaire	Phase 1: Interviews (*n* = 8; patients + caregivers) to generate goals, merged with COPM, FIM, Rivermead. Reviewed via patient focus groups (*n* = 6) and expert panel (*n* = 21; included four OTs). Phase 2: Goal menu used with inpatients (*n* = 130). Patients sorted goal cards (with visuals), rated task difficulty (0–3) and goal importance (1–4).	R: Not OT I: N/A A: OT used all the assessments O: OTs involved in focus groups and expert panel; contributed to development and validation using OT frameworks (e.g., COPM and FIM).	Top rehab priorities were identified as (1) mobility, (2) self‐care and (3) religious practices. There were challenges in mobility and self‐care. Cultural factors influenced goal ranking. The final tool had seven goal domains.
Manee et al. 2017 *Kuwait* Q2 [96] *Prevalence*	Examine use of cognitive assessments, resource availability and obstacles to cognitive rehabilitation practices in Kuwait.	Neurological conditions *n* = 46 (OT = 9, SLP = 13, Psychiatrists = 12, Neurologists = 12)	NR NR	General and private hospitals (10 hospitals; acute care inpatient and outpatient rehab)	Descriptive: 1. MMSE 2. MoCA 3. LOTCA 4. TMT 5. Clock drawing test 6. D2 7. GCS 8. RLA 9. Line‐bisection test 10. COPM 11. FIM 12. BI 13. Interest checklist	N/A	R: First author I: N/A A: N/A O: OT‐specific tools used by 7/9 used LOTCA; 2/9 used COPM. Limited access to resources noted.	Barriers: Lack of continuing education, standardised tools, referral processes; minimal use of occupation‐based assessments; need for translated tools and national guidelines.
Omu et al. 2013 *Kuwait* Q2 [97] *Aim 1: etiologic and risk* *Aim 2: meaning and experience*	Aim 1: Examine link between self‐efficacy and life satisfaction in female first‐stroke patients in Kuwait. Aim 2: To explore health professionals′ views on strategies to boost self‐efficacy in all stroke patients during rehabilitation.	Stroke *n* = 52 Phase 1: *n* = 40 (stroke patients) Phase 2: *n* = 12 (health practitioners)	(55 [10], 33–73) All female	Large rehab hospital (inpatient and outpatient)	Descriptive: 1. GSE	No formal intervention: Phase 2 used interviews to explore practitioner strategies to increase self‐efficacy.	R: Not OT I: N/A A: NR O: Nill	Aim 1: Self‐efficacy strongly correlated with life satisfaction. Aim 2: Key strategies included motivation, education, recognising change, quality therapy and client‐centred goals, with cultural and motivational factors also influencing outcomes.
Shamir, et al. 2024 *Israel* Q1 [98] *Aim 1: Meaning and experience* *Aim 2: Etiologic and risk*	Aim 1: To explore older adults′ experiences and satisfaction with TECH after MCI self‐training; and Aim 2: Links to cognitive change and adherence.	MCI *n* = 14	Median: 73.5 (IQR; 72–76.7) Range: (65–82) (F: *n* = 6, 42.8%; M: *n* = 8, 57.1%)	Community‐dwelling (outpatient)	T1 and T2: 1. MoCA	N/A	R: First and last authors I: N/A A: OT used MoCA O: Moderated participant focus groups using a semistructured guide; two TECH facilitators served as note‐takers/cofacilitators in a separate group.	Aim 1: Satisfaction aligned with perceived gains; three themes identified memory concerns, home self‐training motivating but tech limited. Aim 2: Group sessions valued for support and learning.

Other design *n* = 5								
Al‐Senani et al. 2019 *Saudi Arabia* Q1 [99] *Prognostic*	Forecast stroke incidence and apply international staffing guidelines to estimate future staffing needs and costs over 10 years.	Stroke N/A	NR NR	National stroke healthcare workforce (acute stroke inpatient units and inpatient rehab units)	None	Modelled workforce needs in stroke care: hyperacute stroke units (HASUs), acute stroke units (ASUs) inpatient rehab; staffing ratios adapted; current and projected staff requirements for neurologists, therapists including OTs calculated; cost estimates made for 10 years staffing.	R: Not OT I: N/A A: N/A O: Nill	Projected 67% increase in ischemic stroke cases: substantial increase in required stroke rehab workforce including 137 full‐time equivalents FTE OTs: OT staffing needs estimated: 0.68–0.81 FTE per five beds (HASU/ASU), 0.75 FTE/5 beds (inpatient rehab). Estimated $230 million staffing cost over 10 years.
Ghawami et al. 2020 *Iran* Q3 [100] *Diagnostic*	Adapt international guidelines for cognitive assessment and rehabilitation of traumatic brain injury patients in Iran.	Traumatic brain injury Multidisciplinary expert panel: *n* = 37 Executive committee: *n* = 6	Patients: ≥ 18 years NR	Iranian Ministry of Health and Medical Education—clinical guideline development and standardisation unit NR	Descriptive: 1. GCS	Adaptation of four international guidelines into Iranian context; developed 63 final recommendations including infrastructure needs.	R: Not OT I: N/A A: NR O: Three OTs were members of the expert panel involved in guideline adaptation and final recommendation development.	Four major traumatic brain injury rehab guidelines (e.g., INCOG, the American Congress of Rehabilitation Medicine ACRM); developed 70 initial and 63 final recommendations (24 marked as key). Included two new recommendations on infrastructure. Targeted all six traumatic brain injury recovery stages.
Irvine et al. 2024 *Qatar* Q2 [101] *Diagnostic*	Present a case of Woodhouse–Sakati syndrome (WSS) exhibiting clear phenotypical features despite no neuroimaging findings	Woodhouse–Sakati syndrome *n* = 1	22 years F	Hospital (not specified if public/private) (outpatient)	None	Botox for dystonia, Deep Brain Stimulation (DBS), intensive rehab: PT, OT, SLP.	R: Not OT I: OT focused on grooming and dressing functional tasks. A: N/A O: Nill	First known case of WSS with no abnormalities. Rehab (OT, PT and SLP) improved ADLs and QoL. Highlights importance of genetic testing even with normal neuroimaging.
Lowen, D. 2023 *Oman* Q1 [102] *Prevalence*	Analyse stroke rehab services in Oman, identify system gaps and offer recommendations to enhance rehabilitation standards and care.	Stroke All stroke survivors in Oman	N/A N/A	Stroke rehabilitation (inpatient, outpatient and community‐based rehab)	N/A	N/A	R: Not OT I: N/A A: N/A O: Nill	Stroke rehabilitation in Oman is primarily acute phase; need for expanded workforce and long‐term rehabilitation focus; government aims for holistic stroke treatment.
Landry et al. 2021 *Lebanon* Q1 *#138* [67] Meaning and experience	Examine challenges caused by structural weaknesses in health and rehabilitation systems and propose ways to use crises as opportunities to rebuild stronger, more resilient and rehabilitation‐inclusive health systems.	Catastrophic sudden‐onset disasters Not specified	Not specified Not specified	Emergency, hospital and rehabilitation Acute and postacute rehabilitation	None	N/A	R: Not OT I: N/A A: N/A O: Nill	Explosion damaged hospitals and clinics, reduced rehab access, worsened chronic care and led to shortages in staff, including OT. Highlighted vulnerabilities in lower–middle income (LMIC) health systems and need for rehabilitation‐inclusive rebuilding.

*^a^Eligibility (and nonstandardised) assessments excluded.*

*^b^Only significant outcomes according to authors are reported.*

*^c^Researcher role is only included for the first and/or last author*; citation details for all assessments are in File S1.

Abbreviations: A‐ACS, Arabic‐Activity Card Sort; A‐MPAI‐4, Arabic Mayo‐Portland Adaptability Inventory‐4; A‐PASS‐SR, Arabic Performance Assessment of Self‐Care Skills—Self‐Report; ACS, Activity Card Sort; ADL, Activities of Daily Living; ARAT, Action Research Arm Test; ASIA AIS, American Spinal Injury Association Impairment Scale; BI, Barthel Index; BDI‐II, Beck Depression Inventory‐II; COPM, Canadian Occupational Performance Measure; COPM (Persian version); CTT, Colour Trails Test; D2, D2 Test of Attention; DO‐IT, Dual Overload Interference Test; EDSS, Expanded Disability Status Scale; FAC, Functional Ambulation Categories; FIM, Functional Independence Measure; FM‐UE, Fugl–Meyer Assessment of the Upper Extremity; GAS, Goal Attainment Scaling; GCS, Glasgow Coma Scale; GSE, Generalised Self‐Efficacy Scale; HAB‐v6, Handwriting Assessment Battery, version 6; HADS, Hospital Anxiety and Depression Scale; IADL, Instrumental Activity of Daily Living; ILAT, Israeli Loewenstein Aphasia Test; IPA‐P, Impact on Participation and Autonomy (Persian version); LCVA, left cerebrovascular accident; LOTCA, Loewenstein Occupational Therapy Cognitive Assessment; MCI, mild cognitive impairment; MDT, multidisciplinary team; MMSE, Mini‐Mental State Examination; MoCA, Montreal Cognitive Assessment; MPAI‐4, Mayo‐Portland Adaptability Inventory‐4; MS, multiple sclerosis; N/A, not applicable; NR, not reported; NIHSS, National Institutes of Health Stroke Scale; OT, occupational therapy; OTs, occupational therapists; PBI, Barthel Index (Persian version); PCFS, Post–COVID‐19 Functional Status Scale; PFIM, Functional Independence Measure (Persian version); PTs, physical therapists; QALYs, Quality‐Adjusted Life Years; QoL, Quality of Life; RCTs, randomised controlled trials; RCVA, right cerebrovascular accident; RLA, Rancho Los Amigos; RPE, Borg Rating of Perceived Exertion; RTI‐II, Routine Task Inventory‐II; SCI, spinal cord injury; SCIM‐III, Spinal Cord Independence Measure III; SIS, Stroke Impact Scale; SIS‐16, Stroke Impact Scale‐16; SLP, speech language pathologist; SMART, Specific, Measurable, Attainable, Relevant and Time Related; TMT, Trail Making Test; WMS‐R, Wechsler Memory Scale.

**Table 5:** Qualitative (semistructured or focus group, *n* = 10).

Author Year *Country* Journal ranking Question type	Aim	Participants Sample size (*n*) Age (mean, SD, range) Sex (F [*n*], %; M [*n*], %)	Context (setting)	Intervention overview	OT role(s) ^b^R (researcher) O (other)	^a^Outcome
Alahmari et al. 2023 *Saudi Arabia* Q2 [103] *Meaning and experience*	Investigate how physiatrists, physiotherapists and occupational therapists perceive poststroke fatigue (PSF) and their experiences in managing patients affected by it.	Health professionals *n* = 24 (PT = 8; OT = 8; PMR physicians = 8) NR NR	Public hospital (inpatient and outpatient)	PTs provided individual and group exercise, including endurance training. OTs focused on energy conservation strategies, task simplification and prioritisation. Physicians managed medical comorbidities (e.g., diabetes, depression and sleep hygiene) and prescribed antidepressants when needed. All professionals emphasised energy conservation and education for patients and caregivers.	R: Not OT O: Eight OTs participated in the interviews.	Health professionals had limited awareness and inconsistent practices regarding PSF. Five themes emerged: PSF knowledge, diagnosis/treatment practices, low awareness among professionals and patients, and perceived low priority of PSF, highlighting the need to improve PSF awareness.
Dalvandi et al. 2012 *Iran* Q2 [104] *Meaning and experience*	Aim 1: Investigate experiences of Iranian rehabilitation experts in working with stroke survivors and their families. Aim 2: Gather perspectives from experts on potential improvements to rehabilitation services.	Health professionals *n* = 14 (Neurologists *n* = 2; OT *n* = 2; PT *n* = 1; SLP *n* = 1 Rehabilitation nurses *n* = 2 Social workers *n* = 2 Psychiatrists *n* = 2 Psychologists *n* = 2) NR NR	Private and public clinics (NR)	Clinical‐based rehab considered to be clinic‐based services for community‐dwelling stroke survivors and their families.	R: Not OT O: OTs shared experience and suggestions to improve stroke clinical‐based rehab in Iran.	Aim 1: Core issue: nonintegrated rehab services with six problems: (1) insufficient budget limiting access and recovery; (2) inadequate insurance excluding key therapies; (3) scarce, distant rehab centres; (4) negative public views and cultural barriers; (5) poor care coordination, reliance on students; (6) fragmented services due to lack of teamwork and private clinic competition. Aim 2: Suggested improvements: Integrate rehab into healthcare for better access; establish patient tracking system; raise policymakers′ awareness; provide education for survivors/families; adopt holistic family‐centred care.
Derakhshanrad and Piven 2020 *Iran* Q1 [105] *Meaning and experience*	Clarify the integration of the brain, context and occupation by emphasising the self‐organisation framework that supports neuro‐occupation.	Stroke patients *n* = 11 (42.54 [NR], 24–65) (F: *n* = 4, 36.4%; M: *n* = 7, 63.6%)	Rehab facilities (home, clinic or workplace)	N/A	R: First and last authors O: Collected and analysed data and reviewed emerging matrices.	Study of 11 stroke patients using self‐organisation approach showed interaction between brain, context and occupation via three elements: circular causality (neurological patterns), perturbation (contextual challenges) and occupational participation (daily activities). Findings suggest that this approach facilitated motivation and improved occupational engagement.
Fallahpour et al. 2013 *Iran* Q1 [106] *Meaning and experience*	Explore and describe the lived experience of participation in daily occupations following stroke, and to identify the defining features of this phenomenon in Tehran, Iran.	Stroke patient *n* = 8 (57.12 [NR], 45–63) (F: *n* = 4, 50%; M: *n* = 4, 50%) Health professionals *n* = 5 (OT *n* = 3; neuroscientist *n* = 1; nurse *n* = 1) NR NR	Community (postdischarge from public hospital (participant′s home)	N/A	R: First and last authors O: OT‐led interviews and data analysis	Three themes identified: (1) loss of former activities, roles and autonomy; (2) changed self‐identity, feeling a ‘senseless body’ and rediscovering self through actions and social interaction; (3) feeling disconnected from living life.
Feingold‐Polak et al. 2024 *Israel* No ranking [107] *Meaning and experience*	Investigate the integration of technologies, such as robotics and assistive devices, into stroke rehabilitation in Israel.	Health professionals *n* = 13 (Psychiatrist *n* = 1; OT *n* = 4; PT *n* = 5; Authors with tech experience *n* = 3) NR NR	Public hospital (inpatient, outpatient and community based)	N/A	R: Not OT I: Not applicable A: Not applicable O: OT led focus groups and OT participants shared views	Tech offers objective data, more therapy time and engagement. Barriers: Poor usability, setup time, clinician turnover, cost, attitudes and device location Opportunities: Training, collaboration and incentives. Guidelines: User‐friendly design, hospital support, tech leads, training and patient selection to boost use without replacing therapy.
Omu et al. 2012 *Kuwait* Q4 [108] *Meaning and experience*	Investigate health professionals′ perspectives on the main factors that enhance the QoL for both Kuwaiti and non‐Kuwaiti stroke survivors in Kuwait.	Health professionals *n* = 12 (PT = 7; Nurses = 3; OT = 1; SLP = 1) NR NR	Large rehab centre (inpatient and outpatient)	N/A	R: Not OT I: Not applicable A: Not applicable O: OT (American) interview input; linked loss of function and role to adaptation and QoL in stroke survivors.	Factors identified to increase QoL: (1) functional independence, (2) family support and (3) meaningful activity participation.
Pishkhani et al. 2019 *Iran* Q2 [109] *Meaning and experience*	Investigate factors influencing rehabilitation adherence in Iranian stroke patients.	Stroke patient *n* = 6 (67 [NR], 56–75) (F: *n* = 2, 33.3%; M: *n* = 4, 66.6%) Health professionals *n* = 10 (PT *n* = 3; SLP *n* = 2; OT *n* = 1; Nurse *n* = 2; Psychologist *n* = 1; Neurologist *n* = 1) PT: (35.6 [NR], 30–42) PT: (F: *n* = 2, 66.7%; M: *n* = 1, 33.3%) SLP: (34.5 [NR], 34–35) SLP: (only F: *n* = 2, 100%) Nurse: (37.5 [NR], 36–39) Nurse: (only F: *n* = 2, 100%) OT: 35 years OT: (only M: *n* = 1, 100%) Psychologist: 35 years Psychologist: (only F: *n* = 1, 100%) Neurologist: 37 years Neurologist: (only M: *n* = 1, 100%) Family members *n* = 4 (36 [NR], 22–45) (F: *n* = 2, 50%; M: *n* = 2, (50%)	Public inpatient rehab centres and physical therapy centre. (inpatient and outpatient)	N/A	R: Not OT I: Not applicable A: Not applicable O: OT mentioned the importance of family support for stroke patients.	Four factors identified: (1) Patient related (e.g., disability, beliefs) (2) Team related (e.g., communication) (3) System related (accessibility) (4) Insurance/support related
Sahely et al. 2018 *Saudi Arabia* Q1 [110] *Meaning and experience*	Investigate therapists′ knowledge of the Canadian Best Practice Recommendations for Stroke Care (CBPRSC) related to upper extremity rehabilitation poststroke, along with the barriers and facilitators affecting their implementation.	Stroke patients NR	NR (NR)	N/A	R: Not OT O: OTs shared experiences implementing the CBPRSC.	Therapists found some CBPRSC terms unclear, affecting implementation. Key barriers/facilitators were patient factors, therapist experience, context, resources, roles and guideline views. Canadian and Saudi therapists reported similar challenges, showing consistency across cultures.
Shirozhan et al. 2022 *Iran* Q1 [111] *Meaning and experience*	Examine the experiences of nurses and rehabilitation team members regarding the barriers and facilitators to providing nursing care for patients with disabilities in a rehabilitation hospital.	Health professionals *n* = 16 (OT *n* = 1; Nurses *n* = 12; PMR physician *n* = 1; Nurse assistant *n* = 1; NR *n* = 1) NR NR MS patient *n* = 1 NR NR Caregiver *n* = 1 NR NR	Rehab hospital, (inpatient)	N/A	R: Not OT O: Nill	Three themes on barriers and facilitators of rehabilitation nursing care: (A) Nurse related: Limited rehab training, emotional burden, mentoring improves care and professional communication aids teamwork. (B) Work environment: Error‐focused evaluations reduce motivation, staff shortages and long shifts limit care, inadequate facilities and poor design hinder care, specialised units improve safety and education. (C) Patient/caregiver related: patient cooperation and adaptation affect progress; untrained formal caregivers increase nurses′ workload.
Watkins et al. 2021 *United Arab Emirates* Q1 [112] *Meaning and experience*	Explore perspectives and experiences of stroke rehabilitation experts from 12 countries on the meaning and implications of upscaling rehabilitation within their local contexts.	Health professionals *n* = 12 (Neurologist *n* = 1; PT *n* = 4 OT *n* = 1; OT/PT *n* = 1; SLP *n* = 1; PRM physicians *n* = 4)	University hospital, public/private rehab centres (inpatient and outpatient)	N/A	R: Not OT O: OT participated in an online in‐depth semistructured interview conducted in English	Stroke rehab priorities varied by income: High‐income countries focused on quality and equitable access; upper–middle on specialist workforce and rural MDT; lower–middle and low income on workforce, infrastructure and training. Upscaling needs include strong political commitment, increased awareness, financial investment, workforce expansion with new roles, improved infrastructure and enhanced community‐based rehab with telerehabilitation and stigma reduction.

*^a^Only significant outcomes according to authors are reported.*

*^b^Researcher role is only included for the first and/or last author*.

Abbreviations: MDT, multidisciplinary team; NR, not reported; N/A, not applicable; OT, occupational therapy; OTs, occupational therapists; PMR, physical medicine and rehabilitation; PT, physical therapist; QoL, Quality of Life; SLP, speech language pathologist.

**Table 6:** Standardised assessments reported in quantitative studies.

(Total identified assessment) Assessment name	Frequency	How used		
		Baseline	Outcome	Descriptive
*(* *n* = 69 *)*				
^b^FIM	12	✓	✓	
⌊FIM—Persian version	1			✓
FM‐UE	11	✓	✓	✓
⌊FM‐LE	1	✓	✓	
BI	9	✓	✓	✓
⌊ BI—Persian version	1			✓
COPM	7	✓	✓	✓
^b^MMSE	6	✓	✓	✓
^b^LOTCA	5	✓	✓	✓
MAS	5	✓	✓	✓
^b^MoCA	4	✓	✓	✓
⌊ MoCA—Hebrew version	1	✓	✓	
B&B	3	✓	✓	
ARAT	3	✓	✓	
GCS	3	✓	✓	✓
ROM **(**goniometer**)**	2	✓	✓	
⌊ AROM	1	✓	✓	
MPAI‐4	1		✓	
⌊ MPAI‐4—Arabic version	2			✓
SIS	2			✓
⌊ SIS‐16	1			✓
WMFT	1	✓	✓	
⌊ Streamlined—WMFT	1	✓	✓	
ACS	1		✓	✓
⌊ ACS—Arabic version				
RTI	1			✓
⌊ RTI‐II	1			✓
^b^Clock drawing test	2			✓
^b^TMT	2			✓
^c^CPT	2			✓
MRS	2	✓	✓	
Motricity Index	2	✓	✓	
NIHSS	2	✓	✓	
ASIA AIS	2			✓
WebNeuro	2	✓	✓	
SCIM‐III	2	✓	✓	
IPA—Persian version	2		✓	✓
PASS‐SR—Arabic version	2			✓
Bells cancellation test	1			✓
D2	1			✓
^b^RLA	1			✓
WMS‐R	1			✓
Beck Depression Inventory	1			✓
^b^Line bisection	1			✓
CTT	1			✓
^b^MET‐HV	1	✓	✓	
NeuroTrax (Mindstream)	1	✓	✓	
^c^ACLS	1			✓
FAI	1	✓	✓	✓
PCFS	1	✓	✓	
Lawton IADL	1			✓
FAC	1	✓	✓	
SIAS	1	✓	✓	
FM	1	✓	✓	
RIC‐FAS	1	✓	✓	
CAHAI	1	✓	✓	
HAB‐v6	1		✓	✓
MAL	1	✓	✓	
FRT	1		✓	
^a^CMSA	1			
^a^9‐HPT	1			
J‐T Test	1	**✓**	✓	
^a^Jamar dynamometer	1			
^a^VAS	1			
^b^MEAMS	1	✓	✓	✓
DEX	1	✓	✓	
ILAT	1			✓
ISS	1	✓	✓	
RPE	1	✓	✓	
EDSS	1			✓
GAS	1			✓
HADS	1			✓
Rivermead life goals questionnaire	1			✓
GSE	1			✓
FSS	1	✓	✓	
SF‐12	1	✓	✓	
AIS‐head	1	✓	✓	
SPIRQ	1	✓	✓	
QOLIBRI	1			✓

*^a^Assessment of protocol study.*

*^b^Cognitive assessment.*

*^c^Functional cognition assessment.* Citation details for all assessments are in File S1.

Abbreviations: A‐ACS: Arabic Activity Card Sort; A‐MPAI‐4, Arabic‐Mayo Portland Adaptability Inventory‐4; A‐PASS‐SR, Arabic‐Performance Assessment of Self‐Care Skills‐Self‐Report; ACLS, Allen Cognitive Level Screen; ACS, Activity Card Sort; AIS‐head, Abbreviated Injury Scale (head); ARAT, Action Research Arm Test; AROM, active range of motion; ASIA AIS, American Spinal Injury Association Impairment Scale; B&B, Box and Block Test; BI, Barthel Index; CAHAI, Chedoke Arm and Hand Activity Inventory; CMSA, Chedoke–McMaster Stroke Assessment; COPM, Canadian Occupational Performance Measure; CPT, Cognitive Performance Test; CTT, Colour Trails Test; D2, D2 Test of Attention; DEX, Dysexecutive Questionnaire; EDSS, Expanded Disability Status Scale; FAC, Functional Ambulation Categories; FAI, Frenchay Activities Index; FIM, Functional Independence Measure; FM‐UE, Fugl–Meyer Assessment of the Upper Extremity; FM‐LE, Fugle–Meyer Assessment of Lower Extremity; FM, The Brunnström–Fugl–Meyer Test; FRT, Functional Reach Test; FSS, Fatigue Severity Scale; GAS, Goal Attainment Scaling; GCS, Glasgow Coma Scale; GSE, General Self‐Efficacy Scale; HAB‐v6, Handwriting Assessment Battery, Version 6; HADS, Hospital Anxiety and Depression Scale; IADL, Instrumental Activity of Daily Living; ILAT, Israeli Loewenstein Aphasia Test; IPA‐P, Impact on Participation and Autonomy, Persian version; ISS, Injury Severity Score; J–T Test, Jebsen–Taylor Hand Function Test; LOTCA, Loewenstein Occupational Therapy Cognitive Assessment; MAL, Motor Activity Log; MAS, Modified Ashworth Scale; MEAMS, Middlesex Elderly Assessment of Mental State; MRS, Modified Rankin Scale; MET‐HV, Multiple Errands Test‐Hospital Version; MMSE, Mini‐Mental State Examination; MoCA, Montreal Cognitive Assessment; 9‐HPT, Nine‐Hole Peg Test; NIHSS, National Institutes of Health Stroke Scale; PCFS, Post–COVID‐19 Functional Status Scale; QOLIBRI, Quality of Life after Brain Injury; ROM, range of motion; RIC‐FAS, Rehabilitation Institute of Chicago Functional Assessment Scale; RLA, Rancho Los Amigos; RPE, Borg Rating of Perceived Exertion; RTI‐II, Routine Task Inventory‐II; SCIM‐III, Spinal Cord Independence Measure III; SF‐12, 12‐Item Short Form Health Survey; SIAS, Stroke Impairment Assessment Set; SIS, Stroke Impact Scale; SPIRQ, Self‐Perceptions in Rehabilitation Questionnaire; S‐WMFT, Streamlined Wolf Motor Function Test; TMT, Trail Making Test; VAS, Visual Analogue Scale; WMFT, Streamlined Wolf Motor Function Test; WMS‐R, Wechsler Memory Scale.

## 3. Results

**Figure 1:**
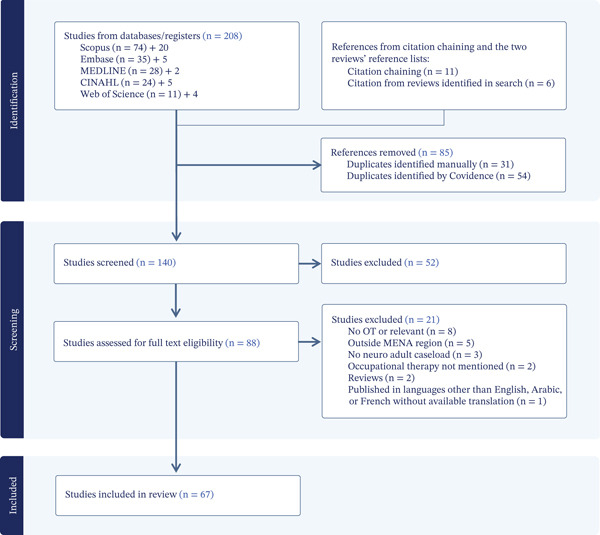
The PRISMA‐ScR flow chart.

The database searches in August 2024 and July 2025, citation tracking of included articles and reviews identified a total of 225 sources. After 85 duplicates were removed, 140 studies were eligible for title/abstract screening. The PRISMA flow chart is presented in Figure 1 (the July 2025 search results are indicated by ‘+’ in the flow chart).

Eleven MENA countries produced neurorehabilitation research, with three countries accounting for approximately 80.5% all output: (Iran [*n* = 23, 34.3%], Israel [*n* = 21, 31.3%] and Saudi Arabia [*n* = 10, 14.9%]). Nine countries had no output: Tunisia, Algeria, Libya, Syria, Yemen, Palestine, Iraq, Bahrain and Mauritania. Most research was produced in the past decade (*n* = 48, 71.6%). Across all studies, stroke (*n* = 48, 64.8%) was the most common neurological condition reported, followed by other neurological conditions, such as encephalitis‐WNF (*n* = 11, 14.8%), TBI (*n* = 6, 8.1%), MS and SCI (*n* = 4, 5.4%, each) and dementia (*n* = 1, 1.3%). For the total of 67 included studies, there were experimental designs (*n* = 19, 28.3%), observational quantitative designs (including cohort, cross‐sectional and other designs, (*n* = 34, 50.7%) and qualitative or multimethods studies (*n* = 14, 20.8%) (see Table 2).

Tables 3, 4 and 5 present a summary of publication characteristics. Research was published in journals ranking Q1 (*n* = 25, 37.3%), Q2 (*n* = 20, 29.8%%), Q3 (*n* = 12, 17.9%) and Q4 (*n* = 7, 10.4%) [113] (journal ranking at the time of study publication). Most research was primary original research (studies) (*n* = 54), using predominantly quantitative approaches, and three were secondary pre‐appraised research and *n* = 3 summaries (see Tables 3, 4 and 5). The original research addressed aims relating to intervention questions (*n* = 24) (see Table 3), prognostic questions (*n* = 10), diagnostic questions (*n* = 11), prevalence questions (*n* = 7), etiologic and risk, and meaning/experience (*n* = 2) [114] (see Tables 4). Ten studies used qualitative approaches to address aims relating to MDT, patients and family meaning/experience. Methodology included semistructured interviews in eight studies, one focus group and one study with interviews and focus groups (see Table 5).

Study samples involved three types of participant groups: (1) patients only, (2) patients together with family members and health professionals and (3) health professionals only. Across all studies, there were 5760 patients, with sample sizes ranging from 1 to 1828 patients, and an age range from 26.9 to 82 years (mean could not be calculated due to insufficient reporting). All patients were recipients of hospital services. In 39 studies (58.2%), there were both male and female patient participants. Only two (2.9%) had male‐only participants, and two (2.9%) had only female participants. Nine (13.4%) studies did not specify any gender, and two (2.9%) specified only one gender (male) and were silent on the gender of the rest of the sample.

Intervention studies reported the service context. The most common context was some sort of ‘rehabilitation’ service (*n* = 10) with a range of different terms being used with the word ‘rehabilitation’ (e.g., rehabilitation centre, rehabilitation clinic or rehabilitation hospital). Other contexts were public hospitals (*n* = 3), private hospitals (*n* = 2) and community‐based clinics (*n* = 2) (e.g., MS clinic and geriatric clinic); other contexts (*n* = 7) were a university and military hospitals, an acute care hospital, an educational hospital, an ADLs clinic, a Red Crescent clinic, a psychiatric hospital and OT laboratory. Two studies did not specify context (see Table 3). Interventions were delivered in inpatient (*n* = 10), outpatient (*n* = 12), home based (*n* = 5) and ‘other’ settings (*n* = 2), such as environment simulation of a city street, and a physical medicine and rehabilitation department (see Table 3).

Across all studies, a total of 252 health professionals participated (ranging from 5 to 46). In most studies (*n* = 66, 98.5%), the age and gender of health professionals were not reported; these were predominantly studies in which the health professionals were service providers or involved in study activities. In those studies where health professionals were participants, only one study presented gender and age information of its participants. In studies where profession was reported, OTs (*n* = 54, 22.2%) were the most common discipline identified, followed by physical therapists (PTs) (*n* = 47, 19.3%), nurses (*n* = 25, 10.2%), speech language pathologists (SPLs) (*n* = 24, 9.8%) neurologists (*n* = 22, 9%), neurosurgeons (*n* = 15, 6.1%), psychologists and psychiatrists (*n* = 14 each, 5.7%), physicians (*n* = 13, 5.3%) and others (such as radiologist) (*n* = 14, 5.7%).

**Figure 2:**
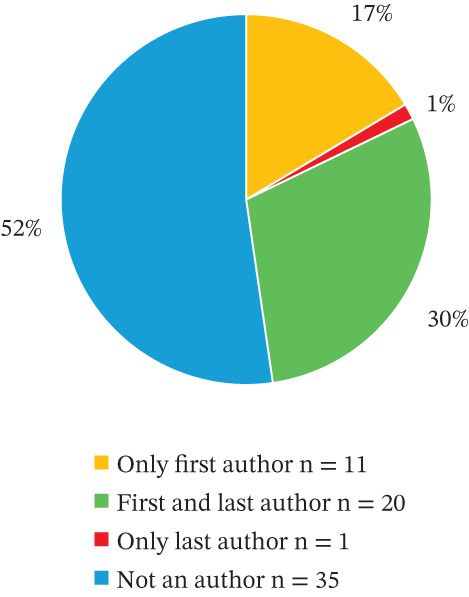
Occupational therapy authorship in the included 67 studies.

OT roles included that of researcher–author. OTs were authors in 32 (48%) of all studies, and of these, 20 (30%) had OTs as first and last authors, 11 (17%) as first author only and one (1%) as a last author (Figure 2). Across the 32 studies that had OT authors, their affiliations were universities only (*n* = 21), university hospitals only (*n* = 1), hospitals only (*n* = 2) and a mix of university and university hospital, hospital and university hospital, university and hospital, and university and commission (*n* = 8). In seven (22%) of the sources, the same OTs were both authors and providers of clinical service, such as assessment and/or treatment. In four (12.5%) studies, different OTs conducted assessments or provided interventions. In the remaining studies, insufficient information was provided to determine whether the author–OTs were involved in assessment or treatment (*n* = 17, 53%). Four (12.5%) studies did not involve assessment or intervention.

OTs collected data in two (20%) of the qualitative studies, using interview methodology. In two (20%) of these instances, the OT collecting data was also an author on the paper. OTs collected data in 30 (93.7%) quantitative studies using standardised (*n* = 60, 68.9%) and nonstandardised (*n* = 20, 22.9%) assessments. In 30 (93.7%) of these instances, the OT collecting data was also an author on the paper. In 17 studies (25.3%), it was not possible to determine who collected the data. OTs administered assessments to evaluate cognition, ADLs, IADLs and motor performance. No home safety assessment was conducted. The other studies either do not describe goal setting or use tools like the Canadian Occupational Performance Measure (COPM) [115] purely as outcome measures rather than as a structured goal‐setting process.

OTs delivered interventions either as part of routine service or as part of a specific study intervention. OT treatment involved ADL training, IADL training, therapeutic exercises, patient education and upper extremity (UE) functional training. OTs also prescribe assistive technology and applied information technology as an intervention approach, which includes virtual reality (VR), such as environmental simulation to cross a street, and computer‐based therapies such as HandTutor [47]. OTs usually delivered services in inpatient and outpatient settings; home visits were rarely used, and telehealth was used for following up discharged patients to check adherence to the training programme and overall well‐being. Additionally, OT used standardised outcome evaluations to measure changes in functional independence, cognitive performance and goal achievement (Tables 3, 4 and 6). The specific neurorehabilitation techniques identified included proprioceptive neuromuscular facilitation (PNF) [116], constraint‐induced movement therapy (CIMT) [117] and Brunnstrom movement therapy (BMT) [118].

Across all quantitative studies, 69 individual published standardised assessments were identified (see Table 6). Most assessments were reported in completed research studies; however, two (2.9%) study protocols were also included, as they reported the use of these standardised assessment measures. Cognition assessments are identified in Table 6 (*n* = 12, 17.3%). There were only five studies that examined validity (construct, concurrent, discriminative and convergent) and reliability of culturally adapted assessments, CPT, IPA‐P, A‐ACS, MPAI‐4 and PFIM [83, 88, 90, 91, 94]. From these studies, only one examined functional cognition assessment (CPT). None of these studies examined cross‐cultural validation. Additionally, studies that used different, updated or translated versions of an original assessment (e.g., Arabic, Persian or Hebrew versions) were identified (see Table 6).

There were 24 original research studies investigating interventions. OTs delivered 22 (91.6%) of the interventions, which ranged from self‐administered programmes and MDT rehabilitation to technology‐assisted OT and VR. Across all intervention studies, only two included goal setting; the other studies did not report a patient‐centred or structured goal framework [51, 58](see Table 3).

Most intervention studies provided replicable protocols that clearly described the inputs, activities and outputs within their programme logic. The inputs referred to the resources and materials used to deliver the intervention, such as therapist expertise, therapy assessment tools and clinical settings. The activities included specific therapeutic actions or processes implemented during sessions, such as task‐oriented training, self‐training, physical exercises, or the frequency and number of sessions. The outputs described the immediate results of these activities, such as skill improvements measured at postintervention. Most intervention studies (*n* = 22, 88%) included enough information to describe the inputs, activities and outputs of the intervention programme logic; three studies did not provide this information: two study protocols that did not produce output [52, 56] and one study using a simulated VR city environment, which did not use standardised assessments [65]. The studies presented problems, including poststroke motor deficits in the upper limbs, post–COVID‐19 disability, mild cognitive impairment and TBI. Interventions focused on cognitive restoration, ADLs independence, functional recovery and reintegration into the community or return home. Among the interventions, 18 studies (72%) used one or more of the standardised assessments to document functional improvements (see Table 3).

Tables 3 and 4 include other study designs, expert opinion papers, economic evaluations, case reports and national guidelines. These designs focused on rehabilitation practice and systems within the MENA region. The expert opinion studies highlighted diverse perspectives such as cognitive rehabilitation frameworks in the context of psychiatric care [71], rehabilitation needs following COVID‐19 [69], workforce and service planning following the 4 August 2020, Beirut explosion [119] and system‐level evaluation of stroke rehabilitation services [102]. Two economic evaluation studies addressed the financial and workforce parameters of rehabilitation: One worked on the cost–benefit analysis of MDT stroke rehabilitation [70], whereas the other estimated national staffing and cost projections for stroke services [99]. The case report study on Woodhouse–Sakati syndrome highlighted the importance of OT for retraining in functional task management [101]. The national guideline study presented trauma‐informed interdisciplinary guidelines for cognitive assessment and reconstruction rehabilitation after a brain injury [100]. Overall, these studies demonstrated the importance of integrating economic, policy and workforce planning perspectives with the clinical outcomes framework to sustain OT practice in the region.

OT researchers explored a wide range of neurorehabilitation topics in the qualitative studies, including (see Table 5) barriers and facilitators to nursing care provision or rehabilitation adherence [109, 111], stroke survivors′ lived experiences [106] and health professionals′ perspectives on quality of life [108]. One study looked at the opinions of therapists regarding the application of best practice guidelines for upper limb rehabilitation [110], and another study examined poststroke fatigue [103]. Additional research examined conceptual approaches like neuro‐occupation [105], scaling up rehabilitation internationally [112], technology integration [107] and community rehabilitation perspectives [104].

### 3.1. Narrative Synthesis of OT Neurorehabilitation in MENA

This results section presents the summary of evidence from the first review of published evidence about OT neurorehabilitation in MENA. This section synthesises this information. Most sources (*n* = 48, 71.6%) were published within the last decade. Three countries, Iran, Israel and Saudi Arabia, accounted for 80.5% of all studies, whereas several other MENA countries had no published research. Studies were published in both regional and international journals, predominantly journals of high quality, showing local importance and wider global recognition.

Several neurological conditions were studied; however, stroke was the most common, with about 64.8%, concentrated on poststroke interventions or outcomes; other conditions, such as dementia, TBI, MS or SCI, were infrequently explored. OTs were substantively involved as authors in 47.7% of publications, typically in leading roles, and nearly all studies involved OTs in their scope or interventions. OTs were most often delivered within MDT alongside PTs, SLPs, nurses and other specialists, mirroring how neurorehabilitation is organised in the region.

There were some experimental design studies, but RCTs (*n* = 11, 16.4%) were limited in number. Most of the studies were observational design, with quantitative studies (*n* = 34, 50.7%) and qualitative or mixed studies (*n* = 14, 20.8%). Most programmes focus on ADLs independence, upper limb or motor recovery, cognitive rehabilitation and community reintegration. They reported improvements in function in both trial and real‐world settings. There are also signs of new ideas, such as VR therapy and computer‐assisted cognitive training, which show promise and are being accepted, mainly when used as a follow‐up at home.

System‐level insights from economic evaluations, disaster‐related studies and national guideline reviews converge. Their focus was to invest in OT workforce development, infrastructure and a more precise role/scope within interdisciplinary pathways, as recommended in studies from Iran [70], Saudi Arabia [99], Lebanon [119] and Oman [102].

Most of the assessments used were standardised assessments (*n* = 69), with some nonstandardised assessments (*n* = 20) to measure motor, cognitive and functional outcomes. Researchers applied assessments that captured performance in daily living, upper limb function, cognition, functional cognition and participation. Interventions targeted physical, cognitive and functional goals that were sometimes supported by technology such as VR or computer‐based training.

Twelve cognition assessments were used (see Table 6). The most commonly used cognitive assessment was the MMSE, followed by the Montreal Cognitive Assessment (MoCA) [120] and the Loewenstein Occupational Therapy Cognitive Assessment (LOTCA) [121]. Cognitive assessments and interventions were included in MENA neurorehabilitation studies; however, the reported use of functional cognition assessments was limited. Only two of the identified cognitive assessments explicitly measured functional cognition, and they were used for descriptive purposes; the term ‘functional cognition’ was not explicitly used to describe them. Practically, this suggests that assessment and intervention of the impact of cognitive impairment on real‐life tasks are currently not part of neurorehabilitation in the MENA region.

## 4. Discussion

This review aimed to characterise the scope of MENA OT neurorehabilitation practice with a secondary focus on cognitive and functional cognitive assessment. Most MENA studies were published in the last 10 years (71.6%), investigating intervention, prevalence, aetiology and experience/meaning questions. Various research designs were used, including RCTs, observational cohort studies, cross‐sectional surveys and qualitative studies using interviews and focus groups. Research visibility is high, with the majority of studies published in top‐tier (Q1/Q2) journals. Among the 20 countries, 11 (55%) had research evidence about OT, with most research being produced by three countries (Iran, Israel and Saudi Arabia)—all countries with > 5 WFOT‐accredited education programmes. Unsurprisingly, most OT authors had affiliations with universities, indicating the importance of university‐based OT programmes and staff in creating the capacity needed to produce OT neurorehabilitation research.

The current review finds that most OT‐led neurorehabilitation research focuses on stroke, whereas other neurological conditions have received little investigation. TBI is among the MENA region′s conditions with high prevalence [122], yet under‐researched in this review, with limited evidence base for TBI rehabilitation in MENA, to support policy and service development [122]. Similarly, dementia is projected to grow by 367% in MENA by 2050 [123]. Yet, the regional research is very limited, and health system capacity is critically low, especially in memory clinics, trained specialists and community support [123]. These patterns underscore that, whereas stroke services are relatively more developed in some MENA countries, other high‐burden conditions, such as TBI and dementia, remain underrepresented in research and poorly served in practice. This highlights the need that MENA OT neurorehabilitation requires diversification of diagnostic focus, region‐specific research and more research information about targeted service development to address the full spectrum of neurological conditions in the region.

Standardised assessments (Table 6) were used as baseline, and/or descriptive and/or outcome measures. Cognition assessments were observed, and there were 12 commonly used cognition assessments; of these, only two were assessments of functional cognition. From these assessments, MMSE, MoCA, LOCTA, MET‐HV and Web‐Neuro were used as outcome measures. The review found that the MMSE is the most used cognitive assessment in MENA neurorehabilitation, followed by the MoCA and LOTCA. This aligns with broader regional data indicating that the MMSE and MoCA are the widely used cognitive assessments to characterise cognitive impairment [124, 125].

One explanation for the dominance of these cognitive assessments is the availability of culturally validated and/or adapted versions in the region′s main languages, for example, Arabic, Persian and Hebrew. For example, the MoCA has validated translations available in Arabic, with an elderly sample in Cairo experiencing MCI, a Persian version validated for individuals with Parkinson′s disease in Iran and a Hebrew version validated for individuals with MCI in Israel [126–128]. This demonstrates the feasibility and importance of culturally relevant evaluations for the region, yet they are still limited, and there is only one functional cognition assessment that is OT specific and has been validated [83].

According to Hammell (2015), most OT theories originate from theorists living in the western/northern world [129]. Accordingly, this could neglect some of the other cultural occupations and ultimately lead to incomplete assessment and improper intervention [36–38, 40]. Since the functional cognition and cognition assessments are essential in OT to assess patients′ cognitive impairments [28, 34], it is also important to translate and culturally adapt them, as well as other theories, assessments and interventions, to be suitable for different regions such as MENA [40, 130]. There was very little information available about whether or not and how these assessments had been translated or adapted for culturally appropriate use in MENA countries. There were only five studies that examined the validity (construct, concurrent, discriminative and convergent) and reliability of culturally adapted assessments. None of these studies examined cross‐cultural validation. This reflects a global trend related to cognitive assessments where rigorous cultural adaptation, including standardised translation, expert review and psychometric validation, is often poorly implemented across regions such as Europe, Asia and Africa [131]. These gaps in adaptation processes can significantly affect validity and reliability, particularly in multilingual and culturally diverse contexts.

Functional cognition is an emerging practice in OT in western countries [31], making it challenging to determine its current scope within MENA OT practice. In this review, the use of functional cognition in the MENA region appears to be limited, mirroring a similar pattern in other western countries [132]. In a large US survey of over 1000 OTs, functional cognition was identified as one of six core domains in adult rehabilitation; yet, only about one‐third of practitioners reported assessing it at every evaluation, with variation across settings [133]. In Australian acute care, OTs working with TBI patients reported positive views of performance‐based functional cognition assessment but continue to rely mainly on nonstandardised observation and interview [44, 134]. In addition, capacity‐building findings from OT in Queensland, Australia, indicate that there is a strong demand for training, particularly in functional cognition [134]. Taken together, limited functional cognition uptake in MENA is consistent with uptake in other western countries; awareness is growing, but confident, routine use will require targeted education, accessible tools, training and proper cultural adaptation.

The absence of home safety assessments, together with the rare use of home visits, was identified in this review, highlighting a critical gap in neurorehabilitation practice in the MENA region. International evidence consistently shows that home visits and home‐based assessments allow OTs to evaluate functional performance, environmental barriers and safety risks that cannot be identified in clinic settings [135, 136]. Strengthening access to home visits and home safety assessments would align regional practice with international rehabilitation standards and improve patient outcomes [20].

Limitations of this review include the absence of an active search for grey literature, as it aimed to map the peer‐reviewed academic literature on OT practice in the MENA region to ensure consistency, methodological transparency and feasibility across multiple countries and languages. Furthermore, in this context, the grey literature is usually difficult to systematically identify and is inconsistently available and described, which may lead to bias. Also, the evidence is concentrated in three countries, limiting regional generalizability; thus, our findings reflect documented OT practice rather than uniform practice across MENA and highlight significant research gaps in underrepresented countries. Lastly, the fact that specific contributions of OTs were sometimes not elaborated in the identified studies, despite their presence on clinical teams in several studies, limits this review′s ability to describe OT roles.

## 5. Conclusion

This scoping review revealed that OTs in MENA are included in neurorehabilitation MDTs. It also showed that OTs independently present and lead research as part of neurorehabilitation services. While the profession is contributing meaningfully to cognitive and functional recovery, particularly in stroke rehabilitation, there remain critical evidence gaps across other neurological conditions, including dementia, TBI, MS and SCI. Despite these gaps, the region has established good foundational work from which to build. Lessons from countries within MENA that have more established neurorehabilitation research infrastructure could help strengthen practice, guide functional cognition assessment development and support capacity building in countries where systems are still emerging, ultimately improving regional quality of care and patient outcomes. Given the prioritisation of functional cognition research in western countries, further research investigating the knowledge and use of functional cognition assessments is also needed, as well as work to culturally adapt and validate them within MENA neurorehabilitation practice.

## 6. Implications for OT Research and Practice

1. OT researchers need to conduct studies that investigate other neurological conditions, such as dementia, TBI, MS and SCI, to provide an evidence base to inform comprehensive neurorehabilitation practice and services in the region.
2. OT awareness of and practice in functional cognition assessment and treatment in MENA neurorehabilitation need further investigation.
3. Most research evidence is generated by university‐affiliated OTs, and more practitioner and practice research is needed, for example, home visit practice, knowledge and use of cognition and functional cognition by OTs.

## Funding

Open access publishing facilitated by the University of Sydney, as part of the Wiley—The University of Sydney agreement via the Council of Australasian University Librarians (CAUL Gold CY26).

## Conflicts of Interest

The authors declare no conflicts of interest.

## Supporting Information

Additional supporting information can be found online in the Supporting Information section.

## Supporting information


**Supporting Information 1.** File S1: List of cognitive and functional cognition assessments. This file provides a comprehensive list of all cognitive and functional cognition assessment tools identified and referenced in the manuscript, including their names and relevant citations, to support transparency and clarity regarding the assessments discussed.

**Supporting Information 2.** File S2: Data extraction template. This file contains the standardised data extraction form developed and used within the Covidence platform to chart study characteristics, participant details, settings, assessment tools (including cognitive and functional cognition measures), and key findings.

**Supporting Information 3.** File S3: PRISMA‐ScR checklist. This file presents the completed Preferred Reporting Items for Systematic Reviews and Meta‐Analyses extension for Scoping Reviews (PRISMA‐ScR) checklist, indicating where each reporting item is addressed within the manuscript.

## Data Availability

The data supporting the results of this study are available from the corresponding author upon request.
