# Supplementary material for: Occupational Therapy Neurorehabilitation Practice in Middle East and North Africa (MENA): A Scoping Review
Source: Occup Ther Int. 2026 May 20;2026:5266195. doi: 10.1155/oti/5266195 (PMC13191774; doi:10.1155/oti/5266195)
Supplement: Supplementary file 1 — Supporting Information 1. File S1: List of cognitive and functional cognition assessments. This file provides a comprehensive list of all cognitive and functional cognition assessment tools identified and referenced in the manuscript, including their names and relevant citations, to support transparency and clarity regarding the assessments discussed. [file OTI-2026-5266195-s002.docx]

**Supplementary file 1. Assessments and Reference List**

- Abbreviated Injury Scale (head) (AIS-head) [1]
- Activity Card Sort (ACS) [2]
- Allen Cognitive Level Screen (ACLS) [3]
- American Spinal Injury Association Impairment Scale (ASIA AIS) [4]
- Arabic-Activity Card Sort (A-ACS) [5]
- Arabic-Mayo Portland Adaptability Inventory-4 (A-MPAI-4) [6]
- Arabic-Performance Assessment of Self-Care Skills Self-Report (A-PASS-SR) [7]
- Barthel Index (BI) [8]; Parsian version [9]
- Beck Depression Inventory-II (BDI-II) [10]
- Borg Rating of Perceived Exertion (RPE) [11]
- Box and Block Test (B&B) [12]
- Canadian Occupational Performance Measure (COPM) [13]; Persian version [14]
- Chedoke Arm and Hand Activity Inventory (CAHAI) [15]
- Chedoke-McMaster Stroke Assessment (CMSA) [16]
- Cognitive Performance Test (CPT) [17]
- Colour Trails Test (CTT) [18]
- D2 Test of Attention (D2) [19]
- Dysexecutive Questionnaire (DEX) [20]
- Expanded Disability Status Scale (EDSS) [21]
- Fatigue Severity Scale (FSS) [22]
- Frenchay Activities Index (FAI) [23]
- Fugl-Meyer Assessment of the Upper Extremity (FM-UE) and FM-LE) [24, 25]
- Functional Ambulation Categories (FAC) [26]
- Functional Independence Measure (FIM) [27]; Persian version [28]
- Functional Reach Test (FRT) [29]
- Generalised Self-Efficacy Scale (GSE) [30]
- Glasgow Coma Scale (GCS) [31]
- Goal Attainment Scaling (GAS) [32]
- Grip and pinch (Jamar dynamometer) [33]
- Handwriting Assessment Battery, version 6 (HAB-v6) [34]
- Hospital Anxiety and Depression Scale (HADS) [35]
- Impact on Participation and Autonomy (IPA) [36]
- Injury Severity Score (ISS) [37]
- Interest Checklist [38]
- Israeli Loewenstein Aphasia Test (ILAT) [39]
- Jebsen-Taylor Hand Function Test (J-T test) [40]
- Lawton instrumental activity of daily living scale [41]
- Line-Bisection [42]
- Loewenstein Occupational Therapy Cognitive Assessment (LOTCA) [43]
- Mayo Portland Adaptability Inventory-4 (MPAI-4) [44]
- Middlesex Elderly Assessment of Mental State (MEAMS) [45]
- Mini-Mental State Examination (MMSE) [46]
- Modified Ashworth Scale (MAS) [47, 48]
- Modified Rankin Scale (MRS) [49]
- Montreal Cognitive Assessment (MoCA) [50]; Hebrew Version [51]
- Motor Activity Log (MAL) [52]
- Motricity Index [53]
- Multiple Errands Test-Hospital Version (MET-HV) [54]
- National Institutes of Health Stroke Scale (NIHSS) [55]
- NeuroTrax (Mindstream) [56, 57]
- Nine-Hole Peg Test (9-HPT) [58]
- Post-COVID-19 Functional Status scale (PCFS) [59]
- Quality of Life after Brain Injury (QOLIBRI) [60]
- Rancho Los Amigos (RLA) [61]
- Range of Motion (ROM) (goniometer) [62]
- Rehabilitation Institute of Chicago Functional Assessment Scale (RIC-FAS) [63]
- Rivermead Life Goals Questionnaire [64, 65]
- Routine Task Inventory (RTI) [66]
- Routine Task Inventory-II (RTI-II) [67]
- Self-Perceptions in Rehabilitation Questionnaire (SPIRQ) [68]
- Spinal Cord Independence Measure III (SCIM-III) [69]
- Streamlined Wolf Motor Function Test (S-WMFT) [70]
- Stroke Impact Scale (SIS) [71]
- Stroke Impact Scale-16 (SIS-16) [72]
- Stroke Impairment Assessment Set (SIAS) [73]
- The Action Research Arm Test (ARAT) [74]
- The bell cancellation test [75]
- The clock drawing test [76]
- Trail Making Test (TMT) [77]
- Visual Analogue Scale (VAS) [78]
- WebNeuro [79]
- Wechsler Memory Scale (WMS-R) [80]
- Wolf Motor Function Test (WMFT) [81]
- 12-Item Short Form Health Survey (SF-12) [82]

References:

1. Carroll CP, Cochran JA, Price JP, Guse CE, Wang MC, editors. The AIS-2005 revision in severe traumatic brain injury: mission accomplished or problems for future research? Annals of Advances in Automotive Medicine/Annual Scientific Conference; 2010.

2. Katz N, Karpin H, Lak A, Furman T, Hartman-Maeir A. Participation in occupational performance: Reliability and validity of the Activity Card Sort. OTJR: Occupation, Participation and Health. 2003;23(1):10-7.

3. Josman N, Katz N. A Problem-Solving Version of the Allen Cognitive Level Test. The American journal of occupational therapy. 1991;45(4):331-8.

4. Maynard Jr FM, Bracken MB, Creasey G, Ditunno Jr JF, Donovan WH, Ducker TB, et al. International standards for neurological and functional classification of spinal cord injury. Spinal cord. 1997;35(5).

5. Hamed R, AlHeresh R, Dahab SA, Collins B, Fryer J, Holm MB. Development of the Arab heritage activity card sort. International Journal of Rehabilitation Research. 2011;34(4):299-306.

6. Hamed R, Tariah HA, Malkawi S, Holm MB. The Arabic version of the Mayo-Portland Adaptability Inventory 4: a validation study. International Journal of Rehabilitation Research. 2012;35(3):243-7.

7. Hamed R, Tariah HA, Jarrar M, Holm M. Development of the Arabic version of the performance assessment of self-care skills. Jordan Medical Journal. 2012;46(3).

8. Fi M. Functional evaluation: the Barthel index. Maryland state medical journal. 1965;14:61-5.

9. Oveisgharan S, Shirani S, Ghorbani A, Soltanzade A, Baghaei A, Hosseini S, Sarrafzadegan N. Barthel index in a Middle-East country: translation, validity and reliability. Cerebrovascular Diseases. 2006;22(5-6):350-4.

10. Ghassemzadeh H, Mojtabai R, Karamghadiri N, Ebrahimkhani N. Psychometric properties of a Persian‐language version of the Beck Depression Inventory‐Second edition: BDI‐II‐PERSIAN. Depression and anxiety. 2005;21(4):185-92.

11. Borg GAV. Psychophysical bases of perceived exertion. Medicine and science in sports and exercise. 1982;14(5):377-81.

12. Mathiowetz V, Volland G, Kashman N, Weber K. Adult norms for the Box and Block Test of manual dexterity. The American journal of occupational therapy. 1985;39(6):386-91.

13. Law M, Baptiste S, McColl M, Opzoomer A, Polatajko H, Pollock N. The Canadian occupational performance measure: an outcome measure for occupational therapy. Can J Occup Ther. 1990;57(2):82-7.

14. Dehghan L, Dalvand H, Pourshahbaz A. Translation of Canadian occupational performance measure and testing Persian version validity and reliability among Iranian mothers of children with cerebral palsy. Journal of Modern Rehabilitation. 2015;9(4):25-31.

15. Barreca S, Gowland C, Stratford P, Huijbregts M, Griffiths J, Torresin W, et al. Development of the Chedoke Arm and Hand Activity Inventory: theoretical constructs, item generation, and selection. Top Stroke Rehabil. 2004;11(4):31-42.

16. Gowland C, Stratford P, Ward M, Moreland J, Torresin W, Van Hullenaar S, et al. Measuring physical impairment and disability with the Chedoke-McMaster Stroke Assessment. Stroke. 1993;24(1):58-63.

17. Burns T, Mortimer JA, Merchak P. Cognitive Performance Test: a new approach to functional assessment in Alzheimer's disease. Journal of geriatric psychiatry and neurology. 1994;7(1):46-54.

18. D’Elia LF, Satz P, Uchiyama CL, White T. Color Trails Test: Professional Manual: Psychological Assessment Resources; 1996.

19. Brickenkamp R, Zilmer E. d2 test of attention. 1998.

20. Wilson BA, Evans JJ, Emslie H, Alderman N, Burgess P. The development of an ecologically valid test for assessing patients with a dysexecutive syndrome. Neuropsychological rehabilitation. 1998;8(3):213-28.

21. Kurtzke JF. Rating neurologic impairment in multiple sclerosis: an expanded disability status scale (EDSS). Neurology. 1983;33(11):1444-.

22. Valko PO, Bassetti CL, Bloch KE, Held U, Baumann CR. Validation of the fatigue severity scale in a Swiss cohort. Sleep. 2008;31(11):1601-7.

23. Schuling J, De Haan R, Limburg Mt, Groenier K. The Frenchay Activities Index. Assessment of functional status in stroke patients. Stroke. 1993;24(8):1173-7.

24. Fugl-Meyer AR, Jääskö L, Leyman I, Olsson S, Steglind S. A method for evaluation of physical performance. Scand J Rehabil Med. 1975;7(1):13-31.

25. Gladstone DJ, Danells CJ, Black SE. The Fugl-Meyer assessment of motor recovery after stroke: a critical review of its measurement properties. Neurorehabilitation and neural repair. 2002;16(3):232-40.

26. Holden MK, Gill KM, Magliozzi MR, Nathan J, Piehl-Baker L. Clinical Gait Assessment in the Neurologically Impaired Reliability and Meaningfulness. Physical therapy. 1984;64(1):35-40.

27. Keitll R, Granger C, Hamilton B. The functional independence measure: a new tool for rehabilitstion. Adv Clin Rehabil. 1987;1:6-18.

28. Naghdi S, Ansari NN, Raji P, Shamili A, Amini M, Hasson S. Cross-cultural validation of the Persian version of the Functional Independence Measure for patients with stroke. Disabil Rehabil. 2016;38(3):289-98.

29. Duncan PW, Weiner DK, Chandler J, Studenski S. Functional reach: a new clinical measure of balance. Journal of gerontology. 1990;45(6):M192-M7.

30. Schwarzer R, Jerusalem M. Generalized self-efficacy scale. J Weinman, S Wright, & M Johnston, Measures in health psychology: A user’s portfolio Causal and control beliefs. 1995;35(37):82-003.

31. Sternbach GL. The Glasgow coma scale. The Journal of emergency medicine. 2000;19(1):67-71.

32. Turner-Stokes L. Goal attainment scaling (GAS) in rehabilitation: a practical guide. Clinical rehabilitation. 2009;23(4):362-70.

33. Mathiowetz V, Weber K, Volland G, Kashman N. Reliability and validity of grip and pinch strength evaluations. The Journal of hand surgery. 1984;9(2):222-6.

34. McCluskey A, Lannin N. The handwriting assessment battery for adults. Unpublished assessment University of Western Sydney. 2003.

35. Zigmond AS, Snaith RP. The hospital anxiety and depression scale. Acta psychiatrica scandinavica. 1983;67(6):361-70.

36. Cardol M, de Haan RJ, Van den Bos GA, de Jong BA, de Groot IJ. The development of a handicap assessment questionnaire: the Impact on Participation and Autonomy (IPA). Clinical rehabilitation. 1999;13(5):411-9.

37. Stevenson M, Segui-Gomez M, Lescohier I, Di Scala C, McDonald-Smith G. An overview of the injury severity score and the new injury severity score. Injury Prevention. 2001;7(1):10-3.

38. Klyczek JP, Bauer-Yox N, Fiedler RC. The interest checklist: A factor analysis. The American Journal of Occupational Therapy. 1997;51(10):815-23.

39. Schecter I. Israeli Loewenstein Aphasia Test (ILAT). Tel Aviv, Loewenstein Rehabilitation Center. 1965.

40. Rh J. An objective and standardized test of hand function. Arch Phys Med Rehabil. 1969;50:311-9.

41. Chong DK-H. Measurement of instrumental activities of daily living in stroke. Stroke. 1995;26(6):1119-22.

42. Jewell G, McCourt ME. Pseudoneglect: a review and meta-analysis of performance factors in line bisection tasks. Neuropsychologia. 2000;38(1):93-110.

43. Katz N, Itzkovich M, Averbuch S, Elazar B. Loewenstein Occupational Therapy Cognitive Assessment (LOTCA) battery for brain-injured patients: reliability and validity. The American Journal of occupational therapy. 1989;43(3):184-92.

44. Guerrette M-C, McKerral M. Validation of the Mayo-Portland Adaptability Inventory-4 (MPAI-4) and reference norms in a French-Canadian population with traumatic brain injury receiving rehabilitation. Disabil Rehabil. 2022;44(18):5250-6.

45. Yaretzky A, Lif-Kimchi O, Finkeltov B, Karpin H, Turani-Feldman T, Shaked-Bregman Y, et al. Reliability and validity of the “Middlesex Elderly Assessment of Mental State”(MEAMS) among hospitalized elderly in Israel as a predictor of functional potential. Clinical gerontologist. 2000;21(4):91-8.

46. Folstein MF, Folstein SE, McHugh PR. “Mini-mental state”: a practical method for grading the cognitive state of patients for the clinician. Journal of psychiatric research. 1975;12(3):189-98.

47. ASHWORTH B. Preliminary trial of carisoprodol in multiple sclerosis. The practitioner. 1964;192:540-2.

48. Bohannon RW, Smith MB. Interrater reliability of a modified Ashworth scale of muscle spasticity. Physical therapy. 1987;67(2):206-7.

49. Newcommon NJ, Green TL, Haley E, Cooke T, Hill MD. Improving the assessment of outcomes in stroke: use of a structured interview to assign grades on the modified Rankin Scale. Stroke. 2003;34(2):377-8.

50. Nasreddine ZS, Phillips NA, Bédirian V, Charbonneau S, Whitehead V, Collin I, et al. The Montreal Cognitive Assessment, MoCA: a brief screening tool for mild cognitive impairment. Journal of the American Geriatrics Society. 2005;53(4):695-9.

51. Lifshitz M, Dwolatzky T, Press Y. Validation of the Hebrew version of the MoCA test as a screening instrument for the early detection of mild cognitive impairment in elderly individuals. Journal of geriatric psychiatry and neurology. 2012;25(3):155-61.

52. Uswatte G, Taub E, Morris D, Vignolo M, McCulloch K. Reliability and validity of the upper-extremity Motor Activity Log-14 for measuring real-world arm use. Stroke. 2005;36(11):2493-6.

53. Bohannon RW. Motricity index scores are valid indicators of paretic upper extremity strength following stroke. Journal of Physical Therapy Science. 1999;11(2):59-61.

54. Knight C, Alderman N, Burgess PW. Development of a simplified version of the multiple errands test for use in hospital settings. Neuropsychological Rehabilitation. 2002;12(3):231-55.

55. Brott T, Adams Jr HP, Olinger CP, Marler JR, Barsan WG, Biller J, et al. Measurements of acute cerebral infarction: a clinical examination scale. Stroke. 1989;20(7):864-70.

56. Dwolatzky T, Whitehead V, Doniger GM, Simon ES, Schweiger A, Jaffe D, Chertkow H. Validity of a novel computerized cognitive battery for mild cognitive impairment. BMC geriatrics. 2003;3(1):4.

57. Schweiger A, Doniger G, Dwolatzky T, Jaffe D, Simon E. Reliability of a novel computerized neuropsychological battery for mild cognitive impairment. Acta Neuropsychologica. 2003;1(4):407-13.

58. Oxford Grice K, Vogel KA, Le V, Mitchell A, Muniz S, Vollmer MA. Adult norms for a commercially available Nine Hole Peg Test for finger dexterity. The American journal of occupational therapy. 2003;57(5):570-3.

59. Siegerink B, Rohmann JL. Impact of your results: beyond the relative risk. Research and practice in thrombosis and haemostasis. 2018;2(4):653-7.

60. von Steinbüchel N, Wilson L, Gibbons H, Hawthorne G, Höfer S, Schmidt S, et al. Quality of Life after Brain Injury (QOLIBRI): scale development and metric properties. Journal of neurotrauma. 2010;27(7):1167-85.

61. Hagen, Malkmus, Durham. Rancho Los Amigos Levels of Cognitive Functioning Scale (LCFS). 1 ed: Routledge; 2010. p. 42-4.

62. Gajdosik RL, Bohannon RW. Clinical measurement of range of motion: review of goniometry emphasizing reliability and validity. Physical therapy. 1987;67(12):1867-72.

63. Roth EJ, Heinemann AW, Lovell LL, Harvey RL, McGuire JR, Diaz S. Impairment and disability: their relation during stroke rehabilitation. Arch Phys Med Rehabil. 1998;79(3):329-35.

64. Wade DT. Goal planning in stroke rehabilitation: how? Top Stroke Rehabil. 1999;6(2):16-36.

65. McGrath JR, Adams L. Patient-centered goal planning: A systemic psychological therapy? Top Stroke Rehabil. 1999;6(2):43-50.

66. Allen CK. Occupational therapy for psychiatric diseases: Measurement and management of cognitive disabilities. (No Title). 1985.

67. Allen CK, Earhart CA, Blue T. Occupational therapy treatment goals for the physically and cognitively disabled: American Occupational Therapy Association Bethesda, MD; 1992.

68. Ownsworth T, Stewart E, Fleming J, Griffin J, Collier AM, Schmidt J. Development and preliminary psychometric evaluation of the Self-Perceptions in Rehabilitation Questionnaire (SPIRQ) for brain injury rehabilitation. The American Journal of Occupational Therapy. 2013;67(3):336-44.

69. Nitsch KP, Stipp KL. Measurement characteristics and clinical utility of the spinal cord independence measure-III among individuals with spinal cord injury. Arch Phys Med Rehabil. 2016;97(9):1601-3.

70. Wu C-y, Fu T, Lin K-c, Feng C-t, Hsieh K-p, Yu H-w, et al. Assessing the streamlined Wolf Motor Function Test as an outcome measure for stroke rehabilitation. Neurorehabilitation and neural repair. 2011;25(2):194-9.

71. Duncan PW, Wallace D, Lai SM, Johnson D, Embretson S, Laster LJ. The stroke impact scale version 2.0: evaluation of reliability, validity, and sensitivity to change. Stroke. 1999;30(10):2131-40.

72. Duncan P, Lai S, Bode R, Perera S, DeRosa J, Investigators GA. Stroke Impact Scale-16: A brief assessment of physical function. Neurology. 2003;60(2):291-6.

73. Chino N, Sonoda S, Domen K, Saitoh E, Kimura A. Stroke impairment assessment set (SIAS) a new evaluation instrument for stroke patients. The Japanese Journal of Rehabilitation Medicine. 1994;31(2):119-25.

74. Lyle RC. A performance test for assessment of upper limb function in physical rehabilitation treatment and research. International journal of rehabilitation research. 1981;4(4):483-92.

75. Gauthier L, Dehaut F, Joanette Y. The bells test: a quantitative and qualitative test for visual neglect. International journal of clinical neuropsychology. 1989;11(2):49-54.

76. Agrell B, Dehlin O. The clock-drawing test. Oxford University Press; 1998.

77. Reitan RM. Validity of the Trail Making Test as an indicator of organic brain damage. Perceptual and motor skills. 1958;8(3):271-6.

78. Wewers ME, Lowe NK. A critical review of visual analogue scales in the measurement of clinical phenomena. Research in nursing & health. 1990;13(4):227-36.

79. Silverstein SM, Berten S, Olson P, Paul R, Williams LM, Cooper N, Gordon E. Development and validation of a World-Wide-Web-based neurocognitive assessment battery: WebNeuro. Behavior research methods. 2007;39(4):940-9.

80. Wechsler D. A standardized memory scale for clinical use. The Journal of Psychology. 1945;19(1):87-95.

81. Wolf SL, Catlin PA, Ellis M, Archer AL, Morgan B, Piacentino A. Assessing Wolf motor function test as outcome measure for research in patients after stroke. Stroke. 2001;32(7):1635-9.

82. Ware JE, Kosinski M, Keller SD. A 12-Item Short-Form Health Survey: construction of scales and preliminary tests of reliability and validity. Medical care. 1996;34(3):220-33.
