# Supplementary material for: Occupational Therapy Neurorehabilitation Practice in Middle East and North Africa (MENA): A Scoping Review
Source: Occup Ther Int. 2026 May 20;2026:5266195. doi: 10.1155/oti/5266195 (PMC13191774; doi:10.1155/oti/5266195)
Supplement: Supplementary file 2 — Supporting Information 2. File S2: Data extraction template. This file contains the standardised data extraction form developed and used within the Covidence platform to chart study characteristics, participant details, settings, assessment tools (including cognitive and functional cognition measures), and key findings. [file OTI-2026-5266195-s003.docx]

**Supplementary file 2. Data Extraction Template (Covidence Platform)**

**1. General information**

**Title**

**Title of paper/abstract/report that data are extracted from:**

| **Authors:** |  |
| --- | --- |
| **Type of publication:** | ☐ Peer-reviewed research article ☐ Peer-reviewed opinion/evidence-based journal article ☐ Book chapter ☐ Abstract/ a publish conference abstract |
| **Year of publication:** |  |
| **Name of the journal:** |  |
| **Journal quality:** |  |
| **Country in which the study conducted:** | ☐ Kingdom of Saudi Arabia ☐ The United Arab Emirates (UAE) ☐ Qatar ☐ Bahrain ☐ Kuwait ☐ Oman ☐ Yemen ☐ Iran ☐ Iraq ☐ Jordan ☐ Syria ☐ Lebanon ☐ Palestine ☐ Israel ☐ Egypt ☐ Algeria ☐ Tunisia ☐ Libya ☐ Mauritania ☐ Morocco ☐ |

**2. Characteristics of included studies**

| **Methods** |  |
| --- | --- |
| **Aim of the study:** |  |
| **Study design** | ☐ Randomised controlled trial ☐ Non-randomised experimental study ☐ Prospective cohort study ☐ Retrospective cohort study ☐ Cross-sectional study ☐ Case control study ☐ Systematic review ☐ Qualitative study ☐ Prevalence study ☐ Case series ☐ Case report ☐ Diagnostic test accuracy study ☐ Clinical prediction rule ☐ Economic evaluation ☐ Program description ☐ Guideline ☐ Editorial / opinion ☐ Other: |

**Primary neurological diagnosis/ health condition primary**

|  | Percentage % |
| --- | --- |
| Stroke |  |
| TBI |  |
| Dementia |  |
| Multiple Sclerosis |  |
| Parkinson’s Disease |  |
| Other |  |

**Co-morbidities:**  ☐ Cognitive Impairments ☐ Mental Health Disorders ☐ Musculoskeletal Problems ☐ Cardiovascular Issues ☐ Seizure Disorder ☐ Chronic Pain ☐ Sleep Disorder ☐ Diabetes ☐ Arthritis ☐ Other:

4. **In what context and setting was the study conducted?**

| **Context** | ☐ Public Hospital ☐ Private clinic ☐ Community based hospital ☐ University hospital ☐ Military hospital ☐ Other: |
| --- | --- |
| **Setting** | ☐ Inpatient ☐ Outpatient ☐ Other: |
| **What mode of service does the study focus on** | ☐ Clinic visit ☐ Home visit ☐ E-health/tele-rehabilitation ☐ Other: |

**5. Participants**

**Participants (what were the participants’ characteristics)**

**Does the study involve a multidisciplinary team:** ☐ **Yes** ☐ **No** ☐ **Not applicable**

**Patients**

|  | **Age** | **Gender** | **Age of onset** | **Education level** |
| --- | --- | --- | --- | --- |
| **Patients** |  |  |  |  |

**Other patient information:**

**Health practitioners**

|  | **Primary discipline** | **Year of experience** | **Qualifications** |
| --- | --- | --- | --- |
| **Health practitioners** |  |  |  |

**Other health professional information:**

**If OTs explain their role:**

**Information about other participants:**

**Assessments**

| **Did the study used or mentioned standardised assessments?** | ☐ Yes ☐ No ☐ Not applicable |
| --- | --- |

**Cognitive assessments used:** If yes, choose from the following ☐ Montreal Cognitive Assessment (MoCA) ☐ Mini-Mental State Examination (MMSE) ☐ Cognitive Assessment of Minnesota (CAM) ☐ Lowenstein Occupational Therapy Cognitive Assessment (LOTCA)☐ Addenbrooke's Cognitive Examination - III (ACE - III) ☐ Other:

**Functional cognition assessments used:** If yes, choose from the following

☐ Allen Cognitive Level Screen (ACLS) ☐ Executive Function Performance Test (EFPT) ☐ Assessment of Motor and Process Skills (AMPS) ☐ Multiple Errands Test (MET) ☐ Performance Assessment of Self-care Skills (PASS)☐ Weekly Calendar Planning Activity ☐ Complex Task Performance Assessment (CTPA) ☐ Cognitive Performance Test (CPT) ☐ Other:

**Other assessments used (standardised and non-standardised):**

**If the study used an assessment, what assessment was administered by an occupational therapist?**

**Other information about OT:**

**Did the study use an outcome measure?** ☐ Yes ☐ No ☐ Not applicable

If yes, choose from the following.

☐ Barthel Index ☐ Functional Independence Measure (FIM) ☐ Canadian ☐ Occupational Performance Measure Goal Attainment Scaling (GAS) ☐Other

**7. Intervention**

| **Is the study mentioned/ focused on an intervention?** | ☐ Yes ☐ No |
| --- | --- |
| **If YES, Describe the intervention:** |  |
| **Other information about intervention:** |  |

**If not an intervention study, specify type (e.g., guideline, program evaluation, descriptive report):**

| **What were the Author reported findings?** |  |
| --- | --- |
| **Researcher observation:** |  |

**Citation chaining (additional citation):**

**Any other relevant information:**
